# Nuclear lipid droplets in Caco2 cells originate from nascent precursors and *in situ* at the nuclear envelope

**DOI:** 10.1016/j.jlr.2024.100540

**Published:** 2024-04-01

**Authors:** Michael McPhee, Jonghwa Lee, Jayme Salsman, Marinella Pinelli, Francesca Di Cara, Kirill Rosen, Graham Dellaire, Neale D. Ridgway

**Affiliations:** 1 Depts of Pediatrics and Biochemistry & Molecular Biology, Atlantic Research Centre, Dalhousie University, Halifax, Nova Scotia, Canada; 2 Depts of Pathology and Biochemistry & Molecular Biology, Dalhousie University, Halifax, Nova Scotia, Canada; 3 Dept of Microbiology and Immunology, Dalhousie University, Halifax, Nova Scotia, Canada

**Keywords:** Nuclear lipid droplets, CCTα, MTP, phosphatidylcholine, promyelocytic leukemia protein

## Abstract

Intestinal epithelial cells convert excess fatty acids into triglyceride (TAG) for storage in cytoplasmic lipid droplets and secretion in chylomicrons. Nuclear lipid droplets (nLDs) are present in intestinal cells but their origin and relationship to cytoplasmic TAG synthesis and secretion is unknown. nLDs and related lipid-associated promyelocytic leukemia structures (LAPS) were abundant in oleate-treated Caco2 but less frequent in other human colorectal cancer cell lines and mouse intestinal organoids. nLDs and LAPS in undifferentiated oleate-treated Caco2 cells harbored the phosphatidate phosphatase Lipin1, its product diacylglycerol, and CTP:phosphocholine cytidylyltransferase (CCT)α. CCTα knockout Caco2 cells had fewer but larger nLDs, indicating a reliance on de novo PC synthesis for assembly. Differentiation of Caco2 cells caused large nLDs and LAPS to form regardless of oleate treatment or CCTα expression. nLDs and LAPS in Caco2 cells did not associate with apoCIII and apoAI and formed dependently of microsomal triglyceride transfer protein expression and activity, indicating they are not derived from endoplasmic reticulum luminal LDs precursors. Instead, undifferentiated Caco2 cells harbored a constitutive pool of nLDs and LAPS in proximity to the nuclear envelope that expanded in size and number with oleate treatment. Inhibition of TAG synthesis did affect the number of nascent nLDs and LAPS but prevented their association with promyelocytic leukemia protein, Lipin1α, and diacylglycerol, which instead accumulated on the nuclear membranes. Thus, nLD and LAPS biogenesis in Caco2 cells is not linked to lipoprotein secretion but involves biogenesis and/or expansion of nascent nLDs by de novo lipid synthesis.

Fatty acids are a source of energy and substrates for membrane biogenesis in eukaryotic cells that when in excess are stored as triglycerides (TAG) in cytoplasmic lipid droplets (cLDs). These dynamic structures contain a hydrophobic core composed of TAG and cholesteryl esters (CE) surrounded by a monolayer of glycerophospholipids, primarily phosphatidylcholine (PC), with associated enzymes and coat proteins (1). Nuclear LDs (nLDs) have also been identified in hepatocytes and hepatocellular carcinoma cells (2, 3, 4), osteosarcoma U2OS cells (4), yeast (5), and *Caenorhabditis elegans* (6). Generally, nLDs are less abundant than their cytoplasmic counterparts and are more frequently observed in cells that are transformed, stressed, or have genetic mutations that alter fatty acid metabolism (reviewed in (7, 8)).

Due to their limited distribution and localization, nLDs are unlikely to contribute significantly to fatty acid storage and energy production. Rather, nLDs interact with nuclear proteins that affect the cellular response to fatty acid excess. For example, the rate-limiting enzyme in PC synthesis CTP:phosphocholine cytidylyltransferase (CCT)α (9) is recruited and activated on the surface of nLDs leading to increased production of phospholipid required to package TAG into cLDs and lipoproteins (4, 10). nLDs also interact with promyelocytic leukemia (PML) protein to form a unique subpopulation that we have termed lipid-associated PML structures (LAPS) (10). PML is the structural scaffold of PML nuclear bodies (PML NBs) that mediate responses to DNA damage, oxidative stress, apoptosis, and transcription by post-translational modification of >150 associated proteins (11, 12). LAPS that form in response to excess oleate are depleted of canonical PML NB proteins such as small ubiquitin-related modifier (SUMO), death domain-associated protein 6 and SP100 (10), and are one of the several PML substructures that assemble in response to other forms of cellular stress (8). Thus, LAPS are modified PML structures that could have attenuated or modified activity, potentially affecting the stress response to fatty acid excess.

In hepatoma Huh7 cells, nLDs and LAPS arise from endoplasmic reticulum luminal lipid droplets (eLDs) that normally fuse with apoB-containing particles to form very low density lipoprotein (VLDL) (13). Under conditions of ER stress and fatty acid overload, eLDs migrate into the type I nucleoplasmic reticulum (NR) and are released into the nucleoplasm by a PML isoform II-dependent mechanism (4, 13). Microsomal triglyceride transfer protein (MTP) is essential for eLD formation and VLDL secretion in hepatocytes (14). Accordingly, pharmacological inhibition of MTP significantly reduced nLD formation in Huh 7 cells. Once in the nucleoplasm, nascent nLDs associate with lipid biosynthetic enzymes and PML and incorporate fatty acids into TAG (4). A second mechanism involving in situ biogenesis of nLDs at the inner nuclear membrane (INM) was identified in U2OS cells (15) and yeast (5, 16). TAG and phospholipid biosynthetic enzymes CCTα, Lipin1, diacylglycerol acyltransferases (DGAT) 1 and 2, and glycerol 3-phosphate acyltransferases 2 and 4 were observed on nLDs and the INM suggesting that localized TAG and phospholipid synthesis drives nLD assembly and expansion (10, 15). This mechanism for nLD biogenesis could be similar to that for cLDs (1, 17), wherein a TAG lens in the INM bilayer expands and eventually buds into the nucleoplasm. However, nLD biogenesis on the INM is independent of seipin (15) and partially dependent on PML expression in U2OS cells (10). In contrast, lipid biosynthetic enzymes at the INM of yeast promote in situ biogenesis by a mechanism that is seipin-dependent (16).

Besides hepatocytes and U2OS cells, nLDs and LAPS are rarely found in other mammalian cells and tissues. An exception is colon carcinoma Caco2 cells that secrete TAG-rich apoB48-containing chylomicrons and contain nLDs and LAPS with associated CCTα (18). The secretion of apoB-containing lipoproteins by differentiated Caco2 cells involves MTP and eLD precursors (19, 20), suggesting nLD biogenesis could involve eLD precursors. Analysis of Caco2 and other human and rodent intestinal-derived cells and organoids revealed nLDs and LAPS with features like those described in hepatocytes and U2OS cells. However, the appearance of nLDs and LAPS in Caco2 and other intestinal-derived cells did not correlate with MTP expression, chylomicron secretion, or their association with apolipoproteins. Instead, evidence pointed to an in situ mechanism involving expansion of a pre-existing nLDs and LAPS and biogenesis on the INM.

## Materials and methods

### Cell culture and transfection

Caco2 (HTB-37, ATCC), Caco2 CCTα KO, Huh7 (JCRB0403, Japanese Collection of Research BioSources cell bank), SW480 (CCL-228, ATCC) and LS180 (CCL-187, ATCC), HCT116 (CCL-247, ATCC), and DLD1 (21), HT29 (21) cells were cultured in Dulbecco’s modified Eagle’s medium (DMEM) containing 10% fetal bovine serum, penicillin (100 units/ml) and streptomycin (100 μg/ml) at 37°C in a 5% CO_2_ atmosphere. IEC18 (CRL-1589, ATCC) and IECras34 (22) were cultured in αMEM containing 5% fetal bovine serum, glucose (3.6 mg/ml), insulin (12.7 μg/ml), and glutamine (2.9 mg/ml) at 37°C in a 5% CO_2_ atmosphere. HT29 cells were cultured in McCoy’s 5A medium supplemented with 10% fetal bovine serum at 37°C in a 5% CO_2_ atmosphere. Caco2 and CCTα KO cells were differentiated into polarized monolayers on polycarbonate membrane inserts (Transwell, Corning) for 21 days, with media changes every 2–3 days. To induce lipid droplets, cells were incubated with an oleate/bovine serum albumin (BSA) complex (6.6:1 mol/mol, 12.7 mM oleate) at a final concentration of 500 μM (23). The oleate/BSA complex was provided to the apical surface of differentiated Caco2 cells. Cells were treated with the DGAT inhibitors A922500 (iDGAT1, Sigma-Aldrich) (24) and PF-06424439 (25) (iDGAT2, Sigma-Aldrich) or the MTP inhibitor lomitapide (Sigma-Aldrich) dissolved in DMSO.

Caco2 cells seeded on glass coverslips were transfected with plasmids encoding murine Lipin1α-V5 (26) or the nuclear GFP-diacylglycerol (DAG) biosensor GFP-C1(2)δ-2xNLS (Addgene #21216) (10, 27) using a 7:1 (μl/μg) ratio of *Trans*IT-X2 transfection reagent (Mirus Bio) to plasmid DNA. Medium was changed 24 h post-transfection and experimental treatments followed at 48 h.

### Enteroid cultures and immunostaining

Intestinal crypts were isolated from 0 days WT Swiss Webster pups (approved by Dalhousie University protocol number 21-023) using Gentle Cell Dissociation Reagent as described in the STEMCELL technology protocols (https://www.stemcell.com/technical-resources/educational-materials/how-to-isolate-mouse-intestinal-crypts.html). Exceptions to the protocol were that intestines were not cut lengthwise before cutting into 2 mm segments and the volume of wash buffers were reduced due to the smaller amount of tissue. Enteroid crypts were cultured in domes of 1:1 (v/v) DMEM F12 with 15 mM Hepes (pH 7.4) and BSA (1%, w/v):Matrigel (Corning) in IntestiCult Organoid Growth Media (STEMCELL Technologies) for 7–10 days before passaging. Once established, mouse enteroid cultures were passaged between 6 and 10 times before conducting experiments. Mouse enteroids were passaged by vigorously breaking apart Matrigel domes using a pre-wet pipette and Stem Gentle Cell Dissociation Reagent for 1 min, incubated for 10 min at 20°C, and collected by centrifugation for 5 min at 290 *g* at 4°C. The pellet was resuspended in DMEM F12 with 15 mM Hepes (pH 7.4) and BSA (1%, w/v) by gentle pipetting and the crypts were sedimented at 200 *g* for 5 min at 4°C and cultured in domes as described above.

Enteroids were prepared for immunofluorescence confocal microscopy after isolation from Matrigel domes with Stem Gentle Cell Dissociation Reagent for 1 min (described above). This was followed by two 20 min incubations on ice with gentle shaking and pipetting to break up Matrigel fragments. Enteroids were collected by gravity sedimentation for 5 min and fixed in 4% paraformaldehyde solution in PBS with gentle shaking for 25 min. Enteroids were washed twice with PBS containing 5 mM NH_4_Cl, 1% BSA, 0.2% (v/v) TritonX-100, 0.1% (v/v) Tween-20. After collecting by gravity sedimentation, fixed enteroids were incubated in permeabilizing blocking buffer (PBS with 1% BSA, 5% (v/v) TritonX-100) for up to 72 h at 4°C. Enteroids were incubated with CCTα and LMNA/C primary antibodies in immunofluorescence buffer (PBS plus 1% BSA, 0.2% (v/v) TritonX-100, 0.1% (v/v) Tween-20) for 72 h at 4°C with gentle shaking. After two washes with immunofluorescence buffer, a secondary goat anti-rabbit AlexaFluor555 and goat anti-mouse AlexaFluor647 were added for 24 h. BODIPY 493/503 was added for 1 h and Hoechst for 10 min. After washing, enteroids resuspended in MOWIOL 4-88 were mounted on glass slides and imaged using a Leica SP8 microscope equipped with 40X and 63X objective lenses as described below for cultured cells. 3D reconstructions of Z-stacks (10–47 sections) and side-view profiles of mouse were analyzed with LASX and Imaris software to identify nuclear lipid droplets.

### Quantitation of [^3^H]oleate incorporation into triacylglycerol

Caco2 cells were pre-treated with DGAT inhibitors or control solvent for 1 h prior to adding 100 μM [^3^H]oleate/BSA. After 4 h, media was removed, cells were rinsed twice with 2% (w/v) BSA in TBS (20 mM Tris-HCl, pH 7.4 and 150 mM NaCl), and then once with TBS before extracting the total lipid fractions with 3:2 hexanes:isopropanol. The lipid extract was resolved using TLC, and [^3^H]oleate incorporation in TAG and CE was quantified relative to total cell protein.

### Immunoblotting

Cell lysates were harvested from dishes and trans-well plates in SDS lysis buffer (12.5% SDS, 30 mM Tris-HCl, pH 6.8, 12.5% glycerol, 0.01% bromophenol blue, and 2% β-mercaptoethanol), heated at 95°C for 3 min, and sonicated for 7–10 s. Proteins were separated by SDS-PAGE and subsequently transferred to nitrocellulose membranes. Membranes were incubated in Licor Odyssey blocking buffer diluted 5:1 (v/v) with TBS-Tween20 (20 mM Tris-HCl, pH 7.4, 150 mM NaCl, and 0.1% Tween20) prior to incubation with primary antibodies for MTP (Novus, cat# NBP1-62489), OSBP (28), CCTα (29), apoAI (Novus, NBP2-52979SS), and apoCIII (Invitrogen, 701238) overnight at 4°C. β-Actin (Sigma Aldrich, A5441) antibody incubations were for 20 min at 20°C. Following incubation with IRDye 800CW and/or IRDye 680LT-labeled secondary antibodies (LI-COR Biosciences), membranes were scanned using LI-COR Odyssey imaging system and fluorescence emission was quantified using associated software (v3.0).

### Immunofluorescence confocal microscopy and super-resolution with radial fluctuations imaging

Cells cultured on glass coverslips were fixed with 4% (w/v) paraformaldehyde for 15 min. Caco2 and CCTα KO cells were permeabilized with 0.5% (w/v) Triton X-100 in PBS (137 mM NaCl, 2.7 mM KCl, 10 mM Na_2_HPO_4_, and 2 mM KH_2_PO_4_, pH 7.4) for 20 min at 4°C. Differentiated Caco2 cells on trans-well inserts were fixed with 4% (w/v) paraformaldehyde for 30 min followed by permeabilization with 0.5% (w/v) Triton X-100 in PBS for 60 min at 20°C. IEC18, IECras34, Huh7, HT29, HCT116, and SW480 cells were permeabilized with 0.2% Triton X-100 in PBS for 10 min at 4°C. Coverslips and trans-well filters were incubated for 24–48 h at 4°C in PBS with 1% (w/v) BSA (PBS/BSA) with the following primary antibodies: anti-PML (Santa Cruz, sc-377390), anti-lamin A/C (Cell Signaling, 4777), as well as those described in the previous section. Following incubation with AlexaFluor-647 and AlexaFluor-555 secondary antibodies, LDs were stained with BODIPY 493/503 or LipidTox Red diluted to 1:500 in PBS for 30 min at 20°C. Nuclei were stained with Hoechst, and coverslips or trans-well inserts were mounted on slides in MOWIOL 4-88. Coverslips were imaged using a Leica TCS SP8 near super-resolution (Lightning) confocal microscopy set up with 4 solid state lasers (405 nm, 488 nm, 552 nm, and 638 nm), an HC Plan APOCHROMAT CS2 100X/1.4 numerical aperture lens, and LASX software set to the Lightning mode. The channel settings used to capture images were the same for control and treated cells.

cLDs, nLDs, and LAPS in undifferentiated Caco2 and Caco2 KO cells were quantified from single z-slices (0.8–1 μm) using Image J (v1.53) as previously described but with the addition of binary masks for the nucleus (Hoechst) and PML (30). A nuclear mask was generated from Hoechst and CCTα channels, a lipid droplet mask from the BODIPY channel, and a LAPS mask from the PML channel, all of which were converted to binary images. To quantify cLDs, the nuclear mask was used to select regions of the BODIPY mask image to delete nLDs and the Binary Reconstruct plugin and the original BODIPY mask were used to identify cLDs. After applying a Water Shedding function to define cLD edges, they were counted using the Analyze Particles plugin. To quantify nLDs, the cLD BODIPY mask was applied to the original BODIPY mask leaving only nLD BODIPY (nLD BODIPY mask), which were subsequently counted with the Analyze Particles plugin. For LAPS, the PML and nLD BODIPY mask were combined with the Binary Reconstruct plugin to quantify nLD BODIPY associated with PML, which were counted with the Analyze Particles plugin. CCTα-positive BODIPY structures in intestinal-derived cell lines (for example see Fig. 4) were counted manually from images open in the LASX software. The complexity and size of cLDs, nLDs, and LAPS in differentiated Caco2 and CCTα KO cells necessitated a more rigorous quantitation method using Image J (v1.53). Channels were split and the images converted into 8 bit followed by background subtraction, addition of gaussian blur to reduce noise, a threshold from which to generate a binary image for PML and BODIPY channels, and a combined mask of the Hoechst and LMNA/C channels. Because of the large size of the lipid droplets and their tendency to push into the nucleus, additional steps of curation were necessary to process the binary masks for quantitation in ImageJ. Manual curation of BODIPY and nuclear masks was carried out using the ImageJ selection brush tool to improve the accuracy of coverage of both BODIPY and nuclear masks, which were subsequently merged to produce an overlap that allowed for identification of candidate nLDs. This image was converted to RGB, exported from ImageJ, and opened in GIMP (v. 2.10.28). The color selection tool was used to removal of all cytoplasmic BODIPY (green binary objects) leaving only nuclear BODIPY (cyan binary objects), which were converted black and white images, exported into ImageJ, and there converted into a binary mask to create an overlay of the original curated BODIPY mask to subtract the nuclear BODIPY. The remaining BODIPY was counted using the Analyze Particles feature and saved as the cLD count. The nuclear BODIPY binary mask used to subtract the nuclear BODIPY from the total BODIPY was used to quantify nLDs using the Analyze Particles feature. Finally, using the Binary Reconstruct plugin, the PML mask was used as a seed to remove all nuclear BODIPY not associated with PML, leaving behind a portion of the binary mask corresponding to LAPS. For 3D rendering of emerin and LMNA/C localization with LDs, differentiated and undifferentiated Caco2 cells were fixed, permeabilized, and immunostained as described above. Z-stacks were captured using a Leica SP8 LIGHTNING confocal microscope and subsequently imported into Imaris software (9.7.0) for 3D rendering as previously described (31).

**Fig. 1:**
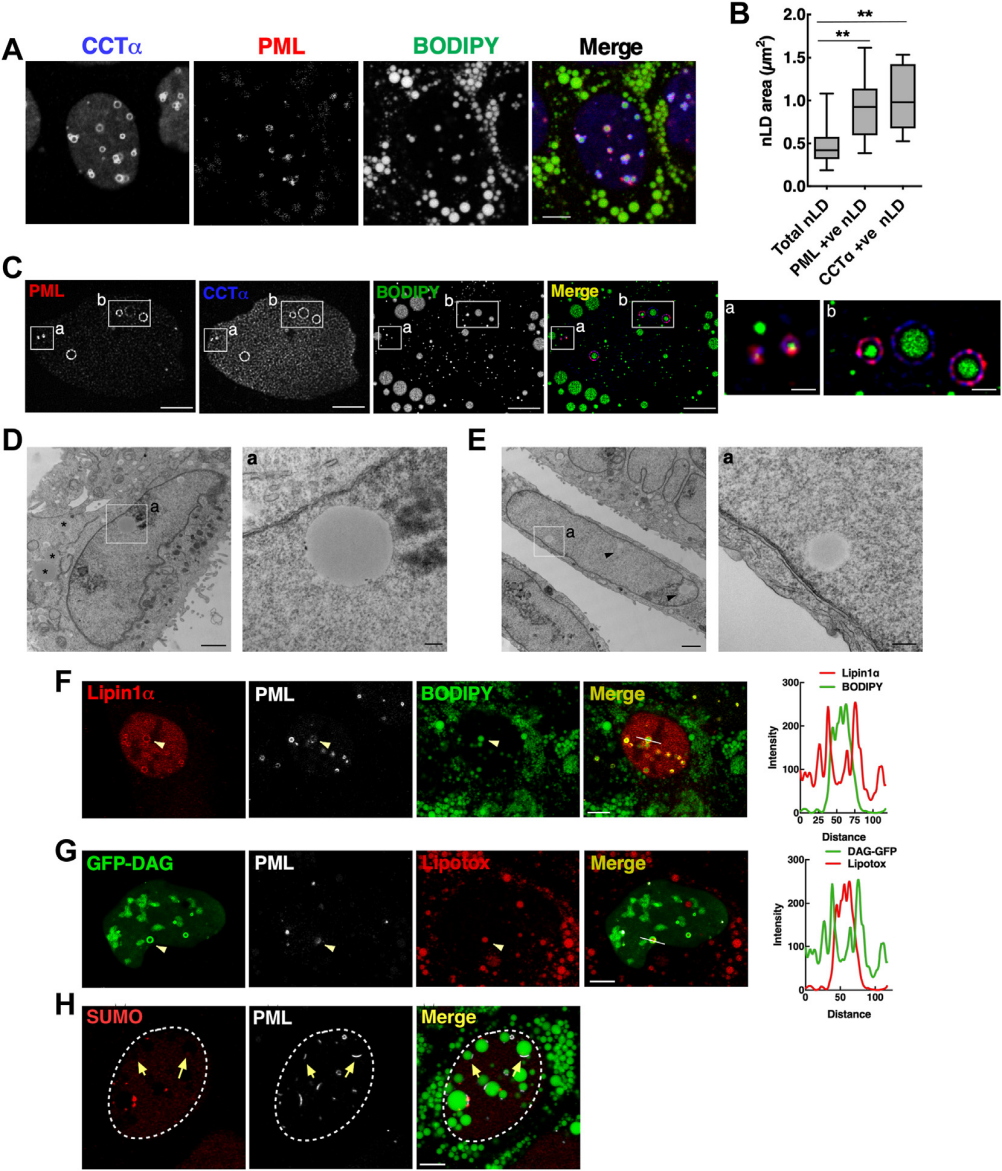
Undifferentiated Caco2 cells contain abundant nLDs and LAPS. A: Caco2 cells were treated with oleate (400 μM) for 24 h, immunostained with CCTα and PML primary followed by AlexaFluor-647 and AlexaFluor-594 secondary antibodies, respectively, and imaged by confocal microscopy. LDs were visualized with BODIPY 493/503 (bar, 5 μm). B: quantification of the average cross-sectional area of total, PML-, and CCTα-positive nLDs. Results show the mean and SD from 6-8 fields of cells (10–15 cells/field) from three separate experiments. Significance was determined by one-way ANOVA and Tukey’s multiple comparison. C: SRRF images of Caco2 cells treated with oleate (400 μM) for 16 h were immunostained for CCTα and PML, BODIPY 493/503 to visualize LDs and imaged by spinning disc confocal microscopy (0.1 μm sections; bar, 5 μm). Enlarged region shown in a and b are boxed (bar, 1 μm). D and E: TEM images of Caco2 cells treated with oleate (500 μM) for 8 h (bar, 1 μm). Enlarged regions are boxed (bar, 200 nm). F: Caco2 cells transiently expressing V5-Lipin1α and treated with oleate (500 μM) for 16 h were immunostained with PML and V5 primary and AlexaFluor-647 and AlexaFluor-555 secondary antibodies, respectively, plus BODIPY 493/503 (bar, 5 μm). The line coordinates for the RGB plot are in the merged image. G: Caco2 cells transiently expressing a GFP-DAG sensor and treated with oleate (500 μM) for 16 h were immunostained for PML plus LipidTox Red (bar, 5 μm). The line coordinates for the RGB plot are in the merged image. H: Caco2 cells were treated with oleate (500 μM for 16 h) and immunostained with SUMO and PML primary followed by AlexaFluor-647 and AlexaFluor-594 secondary antibodies, respectively, plus BODIPY 493/503 (bar, 5 μm). Arrows indicate SUMO-deficient LAPS. ∗∗*P* < 0.01. CCTα, CTP:phosphocholine cytidylyltransferase; LAPS, lipid-associated promyelocytic leukemia structure; nLD, nuclear lipid droplet; PML, promyelocytic leukemia; SUMO, small ubiquitin-related modifier.

**Fig. 2:**
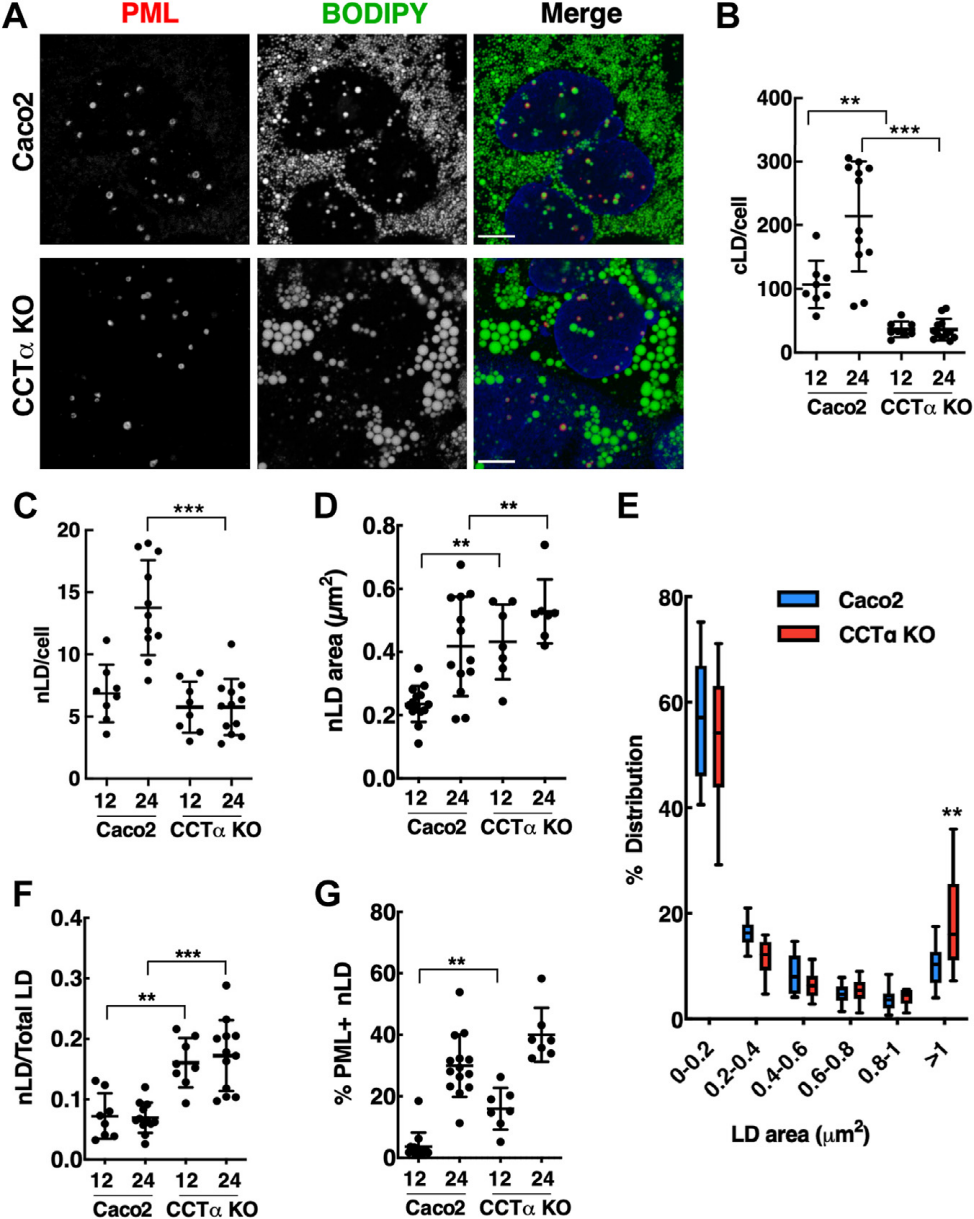
CCTα knockout in undifferentiated Caco2 cells results in fewer and larger nLDs. A: representative confocal images of Caco2 and CCTα-KO cells treated with oleate (400 μM) for 24 h used for quantitation in panels B–G (12 h images not shown). Cells were immunostained with PML and LMNA/C primary followed by AlexaFluor-594 and AlexaFluor-647 secondary antibodies, respectively (bar, 5 μm). LDs were visualized with BODIPY 493/503. B: quantification of cLDs per cell. C: quantification of nLDs per cell. D: the mean area of nLDs. E: binned distribution of nLD area in oleate-treated cells. F: ratio of nLDs to total cLDs plus nLDs. G: percentage of LAPS (PML+ve nLDs) in oleate-treated cells. Results are presented as scatter plots showing the mean and SD from 6-12 fields of cells (10–15 cells/field) from three separate experiments. Panel E is box and whisker plots showing the mean and 5th-to-95th percentile. Significance was determined by two-way ANOVA. ∗∗∗*P* < 0.001, ∗∗*P* < 0.01. CCTα, CTP:phosphocholine cytidylyltransferase; cLD, cytoplasmic lipid droplet; LAPS, lipid-associated promyelocytic leukemia structure; nLD, nuclear lipid droplet; PML, promyelocytic leukemia.

**Fig. 3:**
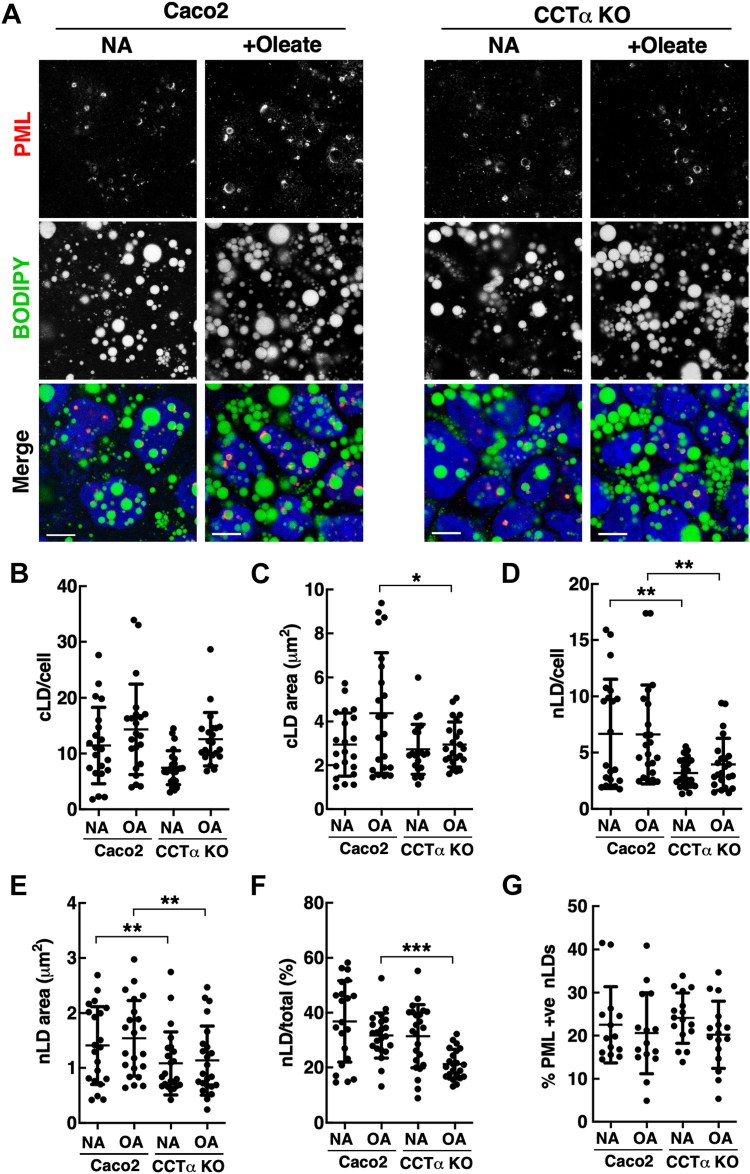
Differentiated Caco2 cells contain a constitutive pool of large nLDs. A: representative confocal image of differentiated Caco2 and CCTα KO cells that received no addition (NA) or oleate (500 μM) for 24 h used for quantitation in panels B–G (bar, 5 μm). Cells were immunostained with PML primary and AlexaFluor-555 secondary antibodies. LDs were visualized with BODIPY 493/503. B: quantification of cLDs per cell. C: mean area of cLDs. D: quantitation of nLDs per cell. E: mean area of nLDs. F: ratio of nLDs to total cLDs plus nLDs. G: percentage of LAPS (PML+ve nLDs) in oleate-treated cells. Results are presented as scatter plots showing the mean and SD from 15-24 fields of cells (60–100 cells/field) from four separate experiments. Significance compared to matched control Caco2 cells was determined by two-way ANOVA. ∗∗∗*P* < 0.001, ∗∗*P* < 0.01, ∗*P* < 0.05. CCTα, CTP:phosphocholine cytidylyltransferase; cLD, cytoplasmic lipid droplet; LAPS, lipid-associated promyelocytic leukemia structure; nLD, nuclear lipid droplet; PML, promyelocytic leukemia.

Super-resolution radial fluctuations (SRRF) imaging is a computational method we employed to generate super-resolution images of Caco2 cells immunostained for CCTα and PML using the following workflow (32, 33). Briefly, wide-fielded images were captured using a 100X Plan-Apochromat (1.46 NA) oil immersion objective lens (Zeiss) on a Marianis microscope (Intelligent Imaging Innovations, 3i) equipped with a SPECTRA III Light Engine (Lumencor) and a Prime BSI back-illuminated scientific complementary metal-oxide semiconductor camera (Teledyne Photometrics). To generate super-resolution images, wide-field images were captured at 10 ms/frame using SlideBook 6 software and exported to ImageJ (version 1.52) in a 16 bit Open Microscopy Environment-Tagged Image File Format. Images were processed using a custom SRRF algorithm (NanoJ-LiveSRRF, Ricardo Henriques, University College London/Francis Crick Institute).

### Transmission electron microscopy

Caco2 cell pellets or cells on trans-well supports were fixed for 2 h with 2.5% (w/v) glutaraldehyde in 0.1 M sodium cacodylate buffer and then in 1% osmium tetroxide for 2 h. Cell pellets were incubated in 0.25% (w/v) uranyl acetate at 4°C overnight, dehydrated, and infiltrated with an Epon araldite resin. The samples were then embedded in 100% Epon araldite resin and placed at 60°C to harden for 48 h. Thin sections of the fixed sample were placed on 300 mesh copper grids and stained with 2% aqueous uranyl acetate and lead citrate. Samples were viewed using a JEOL JEM 1230 Transmission Electron Microscope at 80 kV, and images were captured using a Hamamatsu ORCA-HR digital camera.

### Statistical analysis

GraphPad Prism 9.0 (GraphPad Software Inc) was used for statistical analyses by ANOVA or Students *t* test. Bar and scatter plots show the mean and SD for the number of biological replicates indicated in figure legends with significance denoted as follows: ∗∗∗*P* < 0.001, ∗∗*P* < 0.01, ∗*P* < 0.05.

## Results

### Characterization of nLDs in undifferentiated Caco2 cells

Confocal immunofluorescence microscopy (Fig. 1A) and SRRF imaging of undifferentiated Caco2 cells (Fig. 1C) showed that oleate treatment for 24 h resulted in the formation of abundant nLDs and PML-positive LAPS that were coated with CCTα. CCTα-positive nLDs and LAPS had a greater cross-sectional area (Fig. 1B), perhaps reflecting a more mature droplet. Transmission electron microscopy (TEM) of oleate-treated Caco2 cells revealed that nLDs were not surrounded by the NE but were in proximity to the INM (Fig. 1D, E). Caco2 cells contained type I or II NR that were occasionally in proximity to nLDs (Supplemental Fig. S1A–C). Transiently expressed phosphatidic acid phosphatase Lipin1α (tagged with V5) translocated to the surface of nLDs and LAPS in oleate-treated Caco2 cells (Fig. 1F) where its enzymatic product DAG was also detected using a GFP-DAG biosensor (Fig. 1G). As was the case in U2OS cells (10), LAPS in oleate-treated Caco2 cells were deficient in the canonical PML NB protein SUMO (Fig. 1H, see arrows). Collectively, Caco2 cells contain nLDs and LAPS that have structural features and associated proteins like those identified in Huh7 and U2OS cells (4, 10, 15).

### nLD biogenesis is regulated by CCTα

The knockout of CCTα in Caco2 (30) and other cells (34, 35) results in fewer and larger cLDs due to a deficiency in surface monolayer PC. To assess whether nLD formation was similarly affected by CCTα knockout, undifferentiated Caco2 and CCTα KO cells were incubated with oleate for 12 or 24 h and nLDs and LAPS were visualized with BODIPY493/503 and immunostaining for PML (Fig. 2). As we reported (30), cLDs in oleate-treated CCTα KO cells were significantly reduced and larger than controls (Fig. 2A, B). CCTα KO cells also had significantly fewer nLDs at 24 h but not 12 h of oleate treatments (Fig. 2C). Like their cytoplasmic counterparts, the loss of CCTα expression caused a significant increase in nLD area after oleate treatment for 12 and 24 h (Fig. 2D) and a significant shift in distribution to larger LDs (>1 μm) after oleate treatment for 24 h (Fig. 2E). The large reduction in cLDs in CCTα KO cells (Fig. 2B) caused an increase in the relative proportion of nLDs (Fig. 2F). Interestingly, LAPS were increased in CCTα KO cells at 12 h (Fig. 2G), possibly reflecting reduced competition with CCTα for surface binding to nLDs. To summarize, reduced PC synthesis in undifferentiated CCTα KO cells increased the size but reduced the abundance of cLDs and nLDs, while increasing the prevalence of LAPS.

Differentiated Caco2 cells contain numerous large cLDs whether cultured in the presence or absence of oleate, and CCTα knockout resulted in fewer cLDs with a slight increase in the proportion of very large droplets (30). Since that study excluded nLDs, we undertook a more detailed analysis of the nuclear subfraction in differentiated Caco2 and CCTα KO cells with a well-defined microvilli at the apical surface (Supplemental Fig. S1D). Because cLDs, nLDs, and LAPS in differentiated Caco2 cells were large and frequently impinged the nucleus, they were identified and quantified by immunofluorescence microscopy using three nuclear markers (Hoechst, LMNA/C, and PML). Differentiated Caco2 and CCTα KO cells contained numerous large cLDs, nLDs, and LAPS regardless of the presence of oleate in the culture media (Fig. 3A). TEM of untreated differentiated Caco2 cells confirmed the presence of these large nLDs (Supplemental Fig. S1D, E). The number of cLDs per cell in oleate-treated Caco2 and CCTα KO cells were similar (Fig. 3B), with a minor but significant reduction in the average area of cLDs in CCTα KO cells treated with oleate (Fig. 3C). The number of nLDs per cell was reduced in CCTα KO cells both in the presence and absence of oleate (Fig. 3D). The mean area of nLDs (1–1.5 μm^2^) in control and oleate-treated CCTα KO cells was also reduced compared to controls (Fig. 3E) but overall, nLDs were dramatically larger than in undifferentiated Caco2 cells (0.05–0.1 μm^2^). In addition, nLDs were abundant in both Caco2 and CCTα KO cells, accounting for approximately 30% of total LDs, but only in oleate-treated CCTα-KO cells was the proportion of nLDs reduced significantly relative to matched controls (Fig. 3F). Lastly, LAPS accounted for approximately 20% of nLDs, a proportion that was unaffected by CCTα KO or oleate treatment (Fig. 3G). Collectively, this data set revealed that differentiation of Caco2 cells results in an expanded pool of large nLDs and LAPS that were minimally affected by CCTα knockout or incubation with oleate.

### nLD biogenesis in Caco2 cells is independent of MTP and lipoprotein precursors

**Fig. 4:**
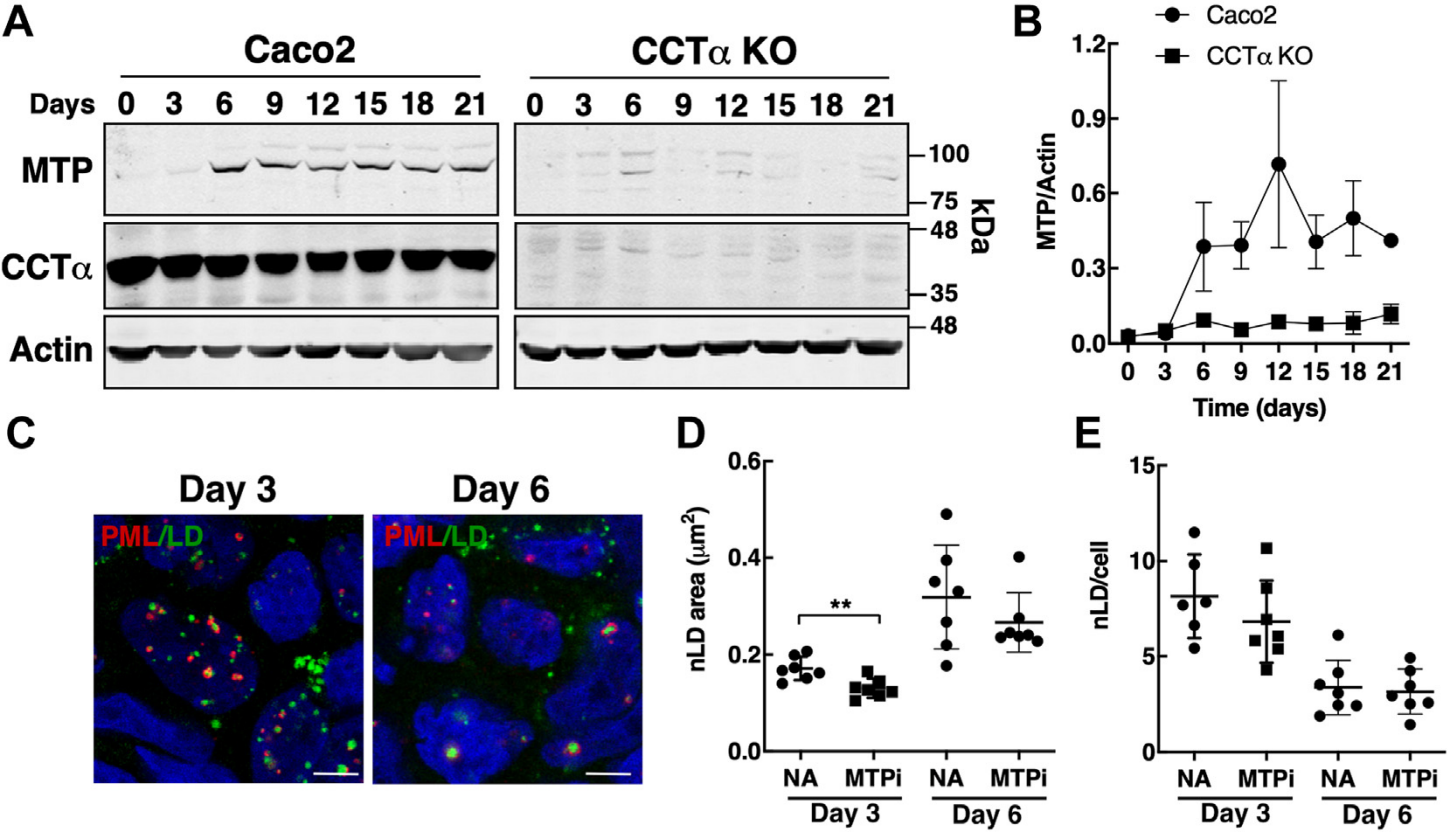
CCTα KO Caco2 cells have reduced MTP expression during differentiation. A: Caco2 and CCTα KO cells were differentiated on trans-well porous supports and at the indicated times were harvested in SDS-PAGE lysis buffer (0 h corresponds to undifferentiated cells cultured on plastic dishes). Lysates were immunoblotted for MTP, CCTα, and actin (load control). B: quantification of MTP expression relative to actin in Caco2 and CCTα KO cells. Results are the mean and SD of three separate experiments. C: confocal images of Caco2 cells at day 3 and 6 of differentiation were immunostained for PML and LDs visualized with BODIPY 493/503 (bar, 5 μm). Nuclei were stained with Hoescht. D: nLD area was quantified in cells differentiated for 3 and 6 cells that received no addition (NA) or MTPi (1 μM). E: nLDs/cell was quantified as described in D. Results are presented as scatter plots showing the mean and SD from seven fields of cells (80–120 cells/field) from a representative experiment. Significance was determined using Students *t* test. CCTα, CTP:phosphocholine cytidylyltransferase; MTPi, MTP inhibitor; nLD, nuclear lipid droplet; PML, promyelocytic leukemia.

Since intestinal epithelial cells contain eLDs (36) and chylomicron secretion by Caco2 cells is MTP-dependent (19, 37), nLDs and LAPS could be derived from the eLD precursors of chylomicrons. As well, MTP expression and chylomicron secretion increases following Caco2 differentiation (38), which could contribute to the proliferation of large nLDs and LAPS. MTP expression in Caco2 cells was almost undetectable in undifferentiated cells (time 0) but increased during the 21-days differentiation on trans-well filters (Fig. 4A, B). MTP expression in CCTα KO cells was significantly reduced by 70%–80% during differentiation, consistent with defective apoB48 chylomicron secretion by those cells (30). We next determined when nLDs and LAPS appeared during the differentiation of Caco2 cells relative to MTP expression. Caco2 cells cultured on transwell supports for 3 days, a time when MTP is not yet induced (Fig. 4B), contained nLDs and LAPS that increased in size by day 6 (Fig. 4C, D). Inclusion of the MTP inhibitor lomitapide (MTPi) during differentiation caused a slight reduction in nLD area on day 3 but had no effect at day 6 when MTP was induced (Fig. 4D). The increased nLD area on day 6 was accompanied by reduction in nLDs/cell indicating a trend toward the large nLDs observed at day 21 (Fig. 3). MTPi had no impact on nLDs/cells again, suggesting a minor role for the eLD/MTP pathway in nLD biogenesis.

**Fig. 5:**
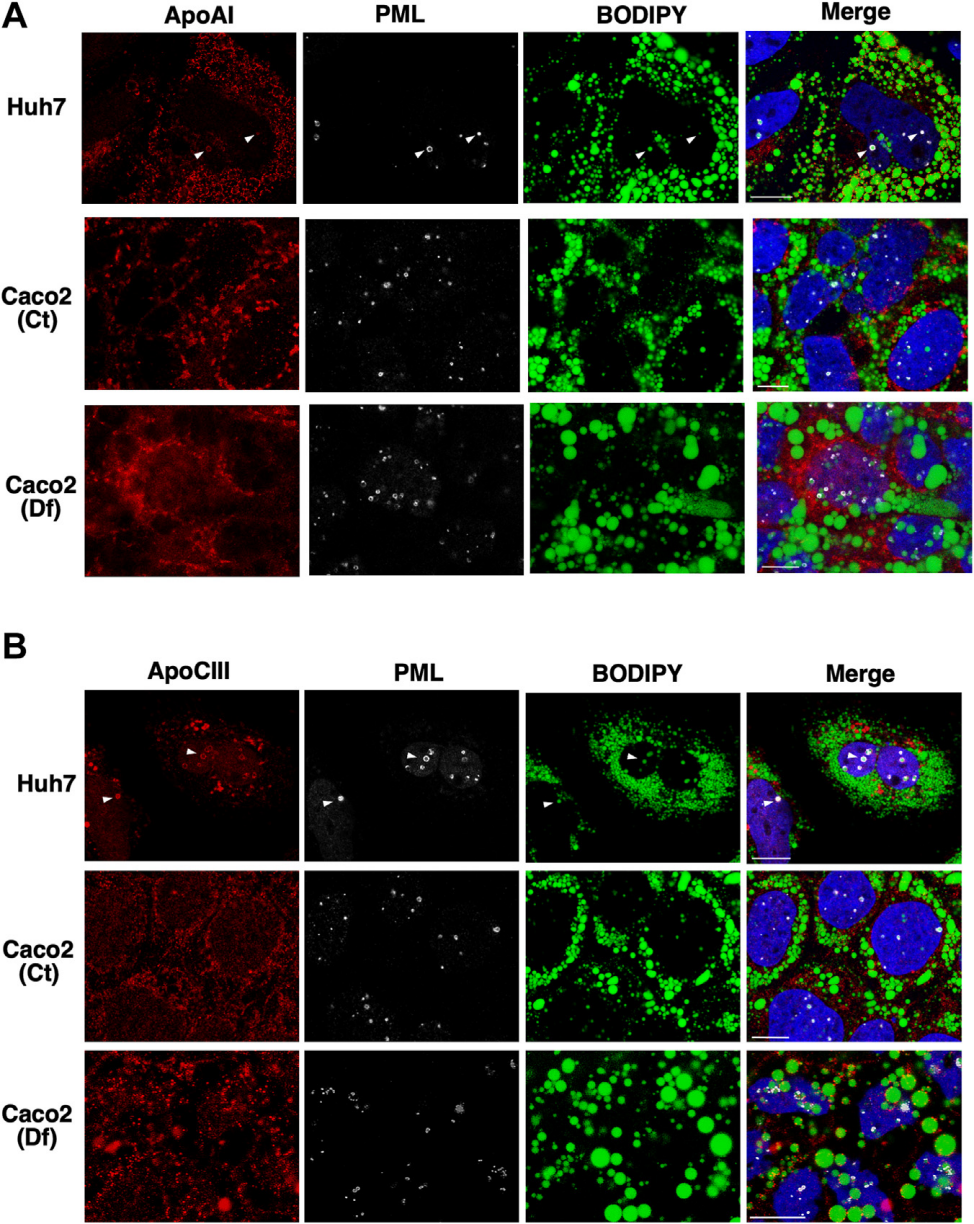
nLDs and LAPS in Caco2 cells do not harbor apoCIII or apoAI. A and B: Huh7 and control undifferentiated (Ct) and differentiated (Df) Caco2 cells were immunostained with antibodies against apoAI (panel A) or apoCIII (panel B) and PML, followed by AlexaFluor-555 and AlexaFluor-647 secondary antibodies. LDs were visualized with BODIPY 493/503 and imaged by confocal microscopy (bar, 10 μm). ApoAI- and apoCIII-positive LAPS are indicated by arrows in the Huh7 cell panels. LAPS, lipid-associated promyelocytic leukemia structure; nLD, nuclear lipid droplet; PML, promyelocytic leukemia.

This conclusion was further tested by examining the association of apolipoproteins with nLDs and LAPS, which in the case of apoE and apoCIII was used as evidence of an eLD origin for nLDs in Huh7 cells (4). Immunofluorescence confocal microscopy confirmed the presence of apoAI and apoCIII on the surface of nLDs and LAPS in Huh7 cells cultured in oleate (indicated by arrows in Fig. 5A, B). ApoAI and apoCIII also appeared on cytoplasmic structures in undifferentiated (Fig. 5A) or differentiated Caco2 cells (Fig. 5B). However, the characteristic ring-like staining pattern for apoAI and apoCIII on nLDs and LAPS observed in Huh7 cells was absent in Caco2 cells suggesting that eLD precursors were not involved.

**Fig. 6:**
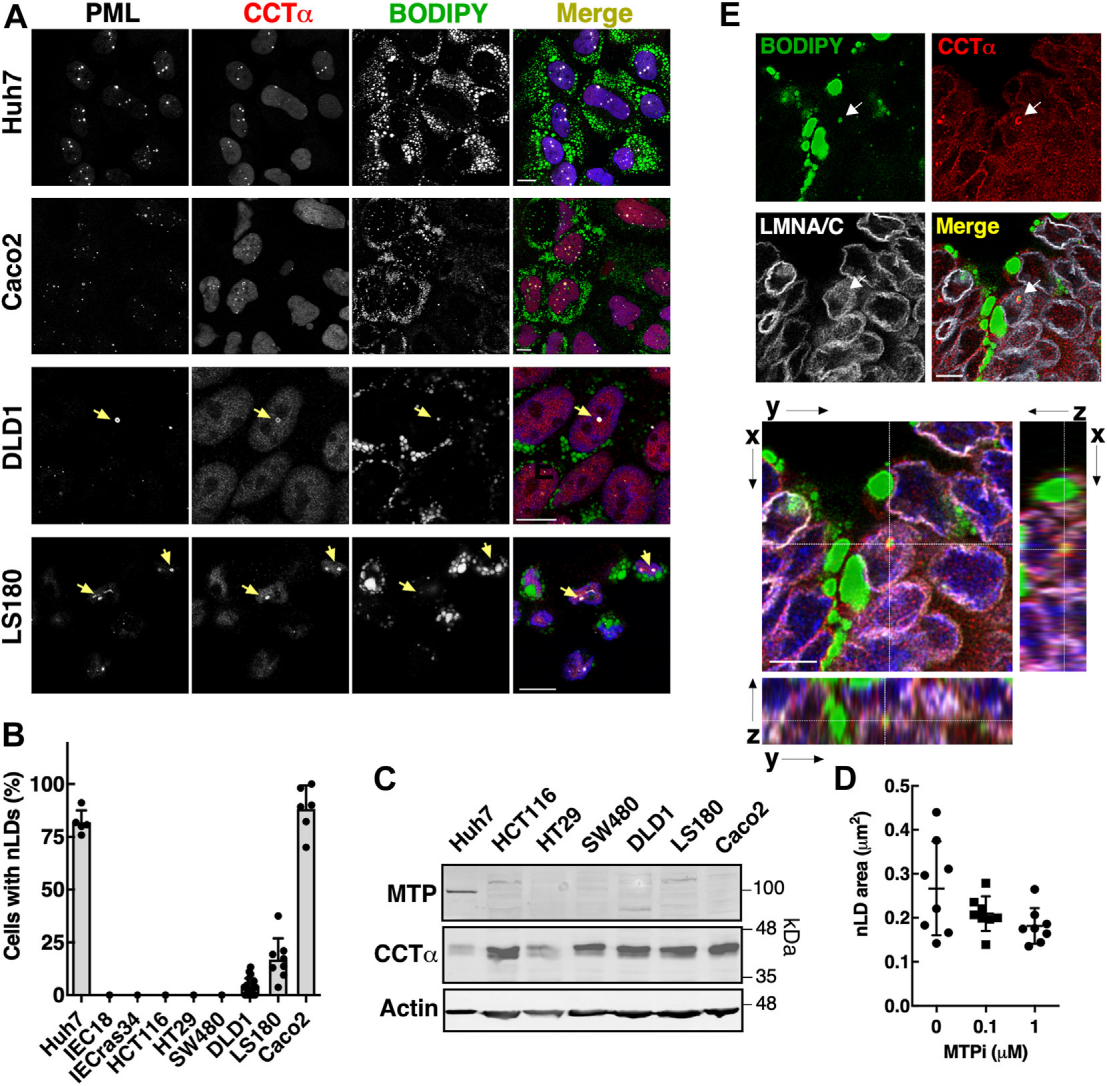
Lack of correlation between MTP expression and frequency of nLDs and LAPS in intestinal-derived cells. A: cells were treated with oleate (500 μM) for 24 h and subsequently immunostained with PML and CCTα primary and AlexaFluor-647 and AlexaFluor-555 secondary antibodies, respectively. LDs were visualized with BODIPY 493/503 and imaged by confocal microscopy (bar, 10 μm). B: percentage of nLD- and LAPS-positive cells from a representative experiment (mean and SD). C: cell lysates were immunoblotted for MTP, CCTα, and actin (load control). D: average nLD cross-sectional area in Caco2 cells treated for 24 h with oleate or oleate plus MTPi. Results are from a representative experiment using 8–10 fields of cells (30–60 cells/field). E: xyz-projection of mouse enteroids immunostained for CCTα and LMNA/C. LDs were visualized with BODIPY 493/503 and nuclei stained with Hoechst. The panels to the right are individual channels from the xy-plane showing the CCTα-positive nLD (indicted by arrow) in the nucleus of an enterocyte (bar, 5 μm). CCTα, CTP:phosphocholine cytidylyltransferase; LAPS, lipid-associated promyelocytic leukemia structure; MTPi, MTP inhibitor; nLD, nuclear lipid droplet; PML, promyelocytic leukemia.

To further investigate the relationship between MTP and nLD/LAPS, immunofluorescence microscopy was used to screen oleate-treated human colorectal cancer cells, as well as rat intestinal epithelial cells (IEC) and transformed IECras34, for the presence of nLDs and LAPS, which was then correlated with MTP expression (Fig. 6A–C). BODIPY-positive nLDs with associated CCTα and/or PML were found in >80% of Caco2 cells and in <20% of DLD1 and LS180 cells (see arrows in Fig. 6A, B). LS180 cells also contained thread-like PML structures. In contrast, CCTα or PML-positive nLDs were not observed in IEC18, IECras34, HCT116, HT29, and SW480 cells (Supplemental Fig. S2). Clusters of BODIPY-positive nLDs were evident in HCT116 and SW480; however, these nLDs were devoid of CCTα and PML, and confocal z-stacks revealed they were cLDs pushed into the nucleus (data not shown). The cell lines shown in Fig. 6B were immunoblotted to determine whether MTP expression was correlated with the presence of nLDs and LAPS (Fig. 6C). None of the human intestinal cell lines had detectable MTP expression compared to Huh7 cells, which contained abundant nLDs and LAPS (4) (Fig. 6A, B). The treatment of Caco2 cells with oleate plus MTPi for 24 h did not significantly affect nLD area (Fig. 6D) or nLDs/cell (results not shown).

The presence of nLDs in mouse enteroids cultured in oleate for 24 h was also examined by confocal immunofluorescence microscopy imaging of CCTα and LMNA/C. Differentiated enterocyte-like cells had cLDs clustered at their basolateral surface at the periphery of the enteroid (Supplemental Fig. S3A). Z-stacks of a region containing enterocytes (see ROI in Supplemental Fig. S3A) contained several LDs with a ring of CCTα and contained within nuclear envelope demarcated by LMNA/C (Fig. 6E and Supplemental Fig. S3B, C). Similar to differentiated Caco2 cells, MTP was expressed in total enteroid cultures and was unaffected by oleate treatment (Supplemental Fig. S3C). Thus, based on a lack of dependence on MTP expression, eLD chylomicron precursors do not play a significant role in nLD and LAPS biogenesis in Caco2 and other intestinal-derived cells.

### A precursor and in situ origin for nLDs and LAPS in Caco2 cells

**Fig. 7:**
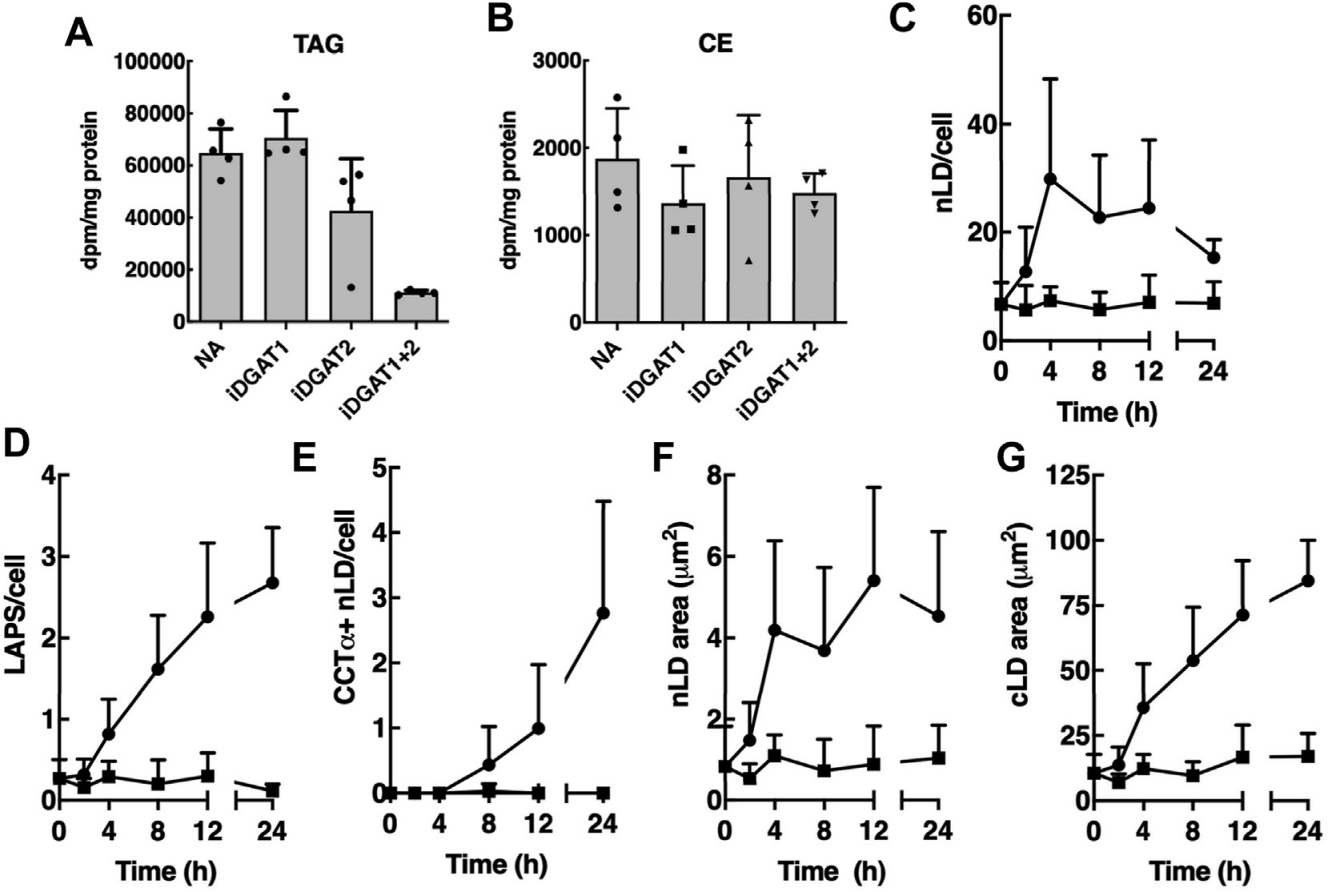
Caco2 cells contain a pool of pre-existing nLDs and LAPS. A and B: Caco2 cells were treated with [^3^H]oleate (100 μM) and iDGAT1 (10 μM), iDGAT2 (10 μM), or both inhibitors (10 μM each) for 4 h. [^3^H]Oleate incorporation into TAG (panel A) and cholesteryl ester (CE, panel B) was measured as described in Materials and Methods. The results are the mean and SD of two experiments carried out in duplicate. C–G: Caco2 cells were treated with oleate (500 μM, •) or oleate plus DGATi (10 μM, ▪). At the indicated times, the nLD/cell (panel C), LAPS/cell (D), CCTα-positive nLDs (panel E), average nLD cross-sectional area (panel F), average cLD cross-sectional (G) were quantified from 15-18 fields of cells (30–60 cells/field) from two experiments. CCTα, CTP:phosphocholine cytidylyltransferase; cLD, cytoplasmic lipid droplet; LAPS, lipid-associated promyelocytic leukemia structure; nLD, nuclear lipid droplet; TAG, triglyceride.

To determine how nLDs and LAPS form in undifferentiated Caco2 cells, immunofluorescence imaging was used to quantify their biogenesis over a 24 h oleate treatment period. Cells were also treated with DGAT1 and DGAT2 inhibitors to determine how blocking TAG synthesis affected nLD and LAPS formation. Treatment of Caco2 cells with inhibitors of DGAT1 or DAGT2 caused weak and variable inhibition of TAG synthesis (Fig. 7A). However, a combination of the two inhibitors (referred to hereafter as DGATi) blocked TAG synthesis by 90% (Fig. 7A) without affecting CE synthesis (Fig. 7B). Untreated Caco2 cells contained 6–8 BODIPY-positive nLDs that increased variably with oleate treatment, eventually doubling by 24 h (Fig. 7C). Untreated cells also contained few LAPS (<0.5/cell) that increased over the treatment period (Fig. 7D). In contrast to PML, CCTα did not associate with nLDs until 8–12 h after oleate treatment (Fig. 7E). The average area of nLDs (Fig. 7F) and cLDs (Fig. 7G) increased after an initial lag. Not unexpectedly, the oleate-dependent increase in nLDs, cLDs, and LAPS was prevented by DGATi. In contrast, pre-existing nLDs and LAPS were unaffected by DGATi treatment for 24 h (Fig. 7C, D), indicating a stable pool of nascent droplets that expand upon exposure of cells to oleate.

**Fig. 8:**
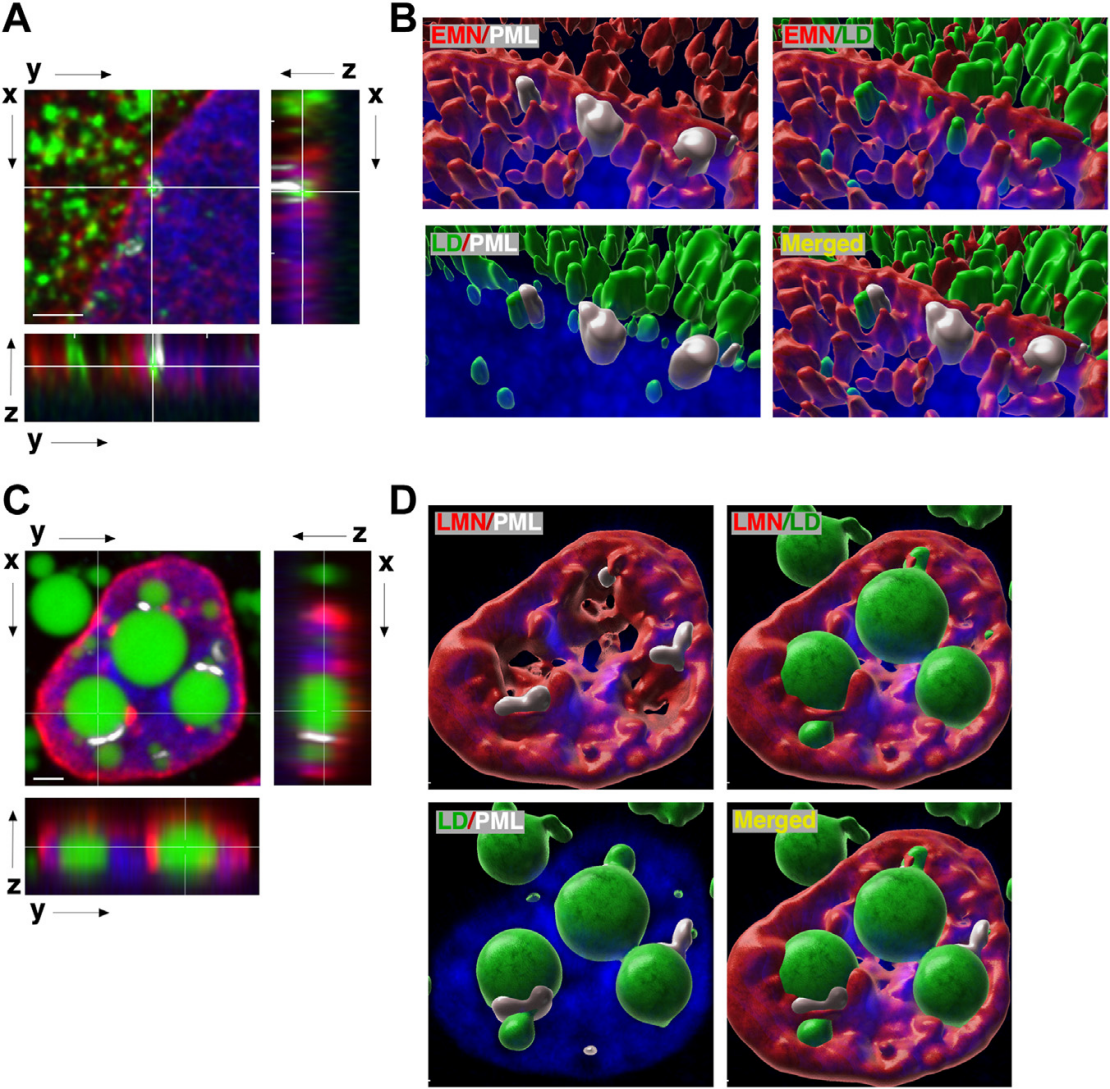
nLDs and LAPS in Caco2 cells associate with the nuclear envelope and nucleoplasmic reticulum. A and B: undifferentiated Caco2 cells treated with oleate (500 μM) for 2 h were fixed and immunostained with emerin and PML primary and AlexaFluor-555 and AlexaFluor-647 secondary antibodies, respectively. Z-stacks of cells containing nLDs and LAPS are shown in panels A (bar, 2 μm). 3D rendering of these projections (panels B) are described in Materials and methods. The 3D images are viewed from the top surface and rotated 90^°^ relative to the x-y plane in panel A. C and D: differentiated Caco2 cells treated with oleate (500 μM) for 24 h were immunostained with LMNA/C and PML primary and AlexaFluor-555 and AlexaFluor-647 secondary antibodies, respectively. Z-stacks in panel C (bar, 2 μm) and 3D images in panel D (rotated slightly left and viewed from the basolateral surface) were generated as described above. LAPS, lipid-associated promyelocytic leukemia structure; nLD, nuclear lipid droplet; PML, promyelocytic leukemia.

Nascent nLDs were visualized by 3D reconstructions of nuclei generated from confocal Z-stacks of undifferentiated Caco2 cells treated with oleate for 2 h. Small nLDs, some with associated PML, were identified in very close proximity to the NE (Fig. 8A). nLDs were often sandwiched between the emerin-positive NE and PML, suggestive of an early stage of biogenesis (Fig. 8B). In contrast, nLDs and LAPS in differentiated Caco2 cells were large (>2 μm in diameter) and had small caps of PML on their surface (Fig. 7C, D, and Supplemental Fig. S4). Since these large nLD and LAPS form during differentiation (Fig. 4) and are not induced by oleate (Fig. 3), it was difficult to assess where they originated. However, small nLDs and LAPS were frequently close to or associated with the NE while larger nLDs and LAPS were in proximity to vertical LMNA/C structures in the interior of the nucleus corresponding to the NR.

**Fig. 9:**
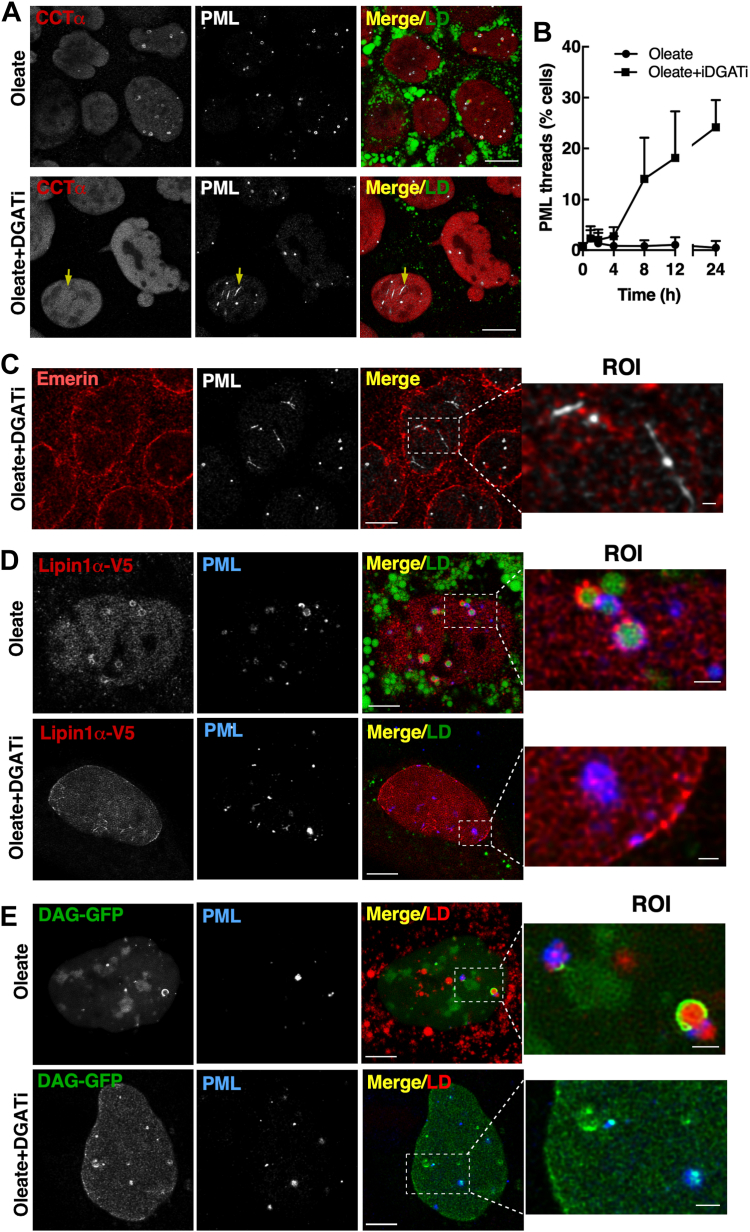
DGAT inhibition in undifferentiated Caco2 cells promotes Lipin1α and DAG accretion at the nuclear envelope. A: Caco2 cells were treated with oleate (500 μM) or oleate plus DGATi (10 μM) for 24 h and immunostained with CCTα and PML primary and AlexaFluor-555 and AlexaFluor-647 secondary antibodies, respectively. LDs were visualized with BODIPY493/503 (bar, 10 μm). B: the frequency of cells positive for PML threads was determined after treatment for the indicated times with oleate (500 μM) or oleate plus DGATi (10 μM). Results are from 15-18 fields of cells (30–60 cells/field) for two experiments. C: Caco2 cells treated as described in panel A were immunostained with emerin and PML primarily and AlexaFluor-555 and AlexaFluor-647 secondary antibodies, respectively (bar, 10 μm; zoom image bar, 1 μm). D: Caco2 cells treated as described in panel A and transiently expressing Lipin1α-V5 were immunostained with V5 and PML primary and AlexaFluor-555 and AlexaFluor-647 secondary antibodies, respectively. LDs were visualized with BODIPY493/503 (bar, 5 μm; zoom image bar, 1 μm). E: Caco2 cells transiently expressing a GFP-DAG biosensor and treated as described in panel A were immunostained with a PML monoclonal and AlexaFluor-647 secondary antibody. LDs were visualized with LipidTox Red (bar, 5 μm; zoom image bar, 1 μm). CCTα, CTP:phosphocholine cytidylyltransferase; nLD, nuclear lipid droplet; PML, promyelocytic leukemia.

Temporal analysis of nLD and LAPS formation and 3D imaging suggested that a pool of small nLDs and LAPS in proximity to the INM were converted to mature droplets during exposure to oleate. Since these nascent nLDs remained static during inhibition of TAG synthesis, we used immunofluorescence microscopy of Caco2 cells treated with oleate plus iDGAT to determine the fate of CCTα, DAG, PML, and Lipin1α that are involved in nLD maturation. Inhibiting nLDs and LAPS biogenesis in oleate- and DGATi-treated cells caused CCTα to remain in the nucleoplasm (Fig. 9A). Under these conditions, PML appeared in punctate nuclear bodies but also in unusual filaments (indicated by arrows in Fig. 9A). Filamentous PML structures in iDGAT-treated cells appeared in 10%–20% of cell by 8 h (Fig. 9B). PML filaments in iDGAT-treated cells did not localize with emerin on the NE or NR (Fig. 9C). Lipin1α-V5 was on nLDs and LAPS in oleate-treated cells but addition of DGATi caused the enzyme to shift to the NE and nucleoplasmic puncta and filaments, which partially colocalized with PML (Fig. 9D). Similar to Lipin1α, iDGAT caused the GFP-DAG biosensor to localize to the NE and cytoplasmic puncta, some of which were also positive for PML (Fig. 9E).

**Fig. 10:**
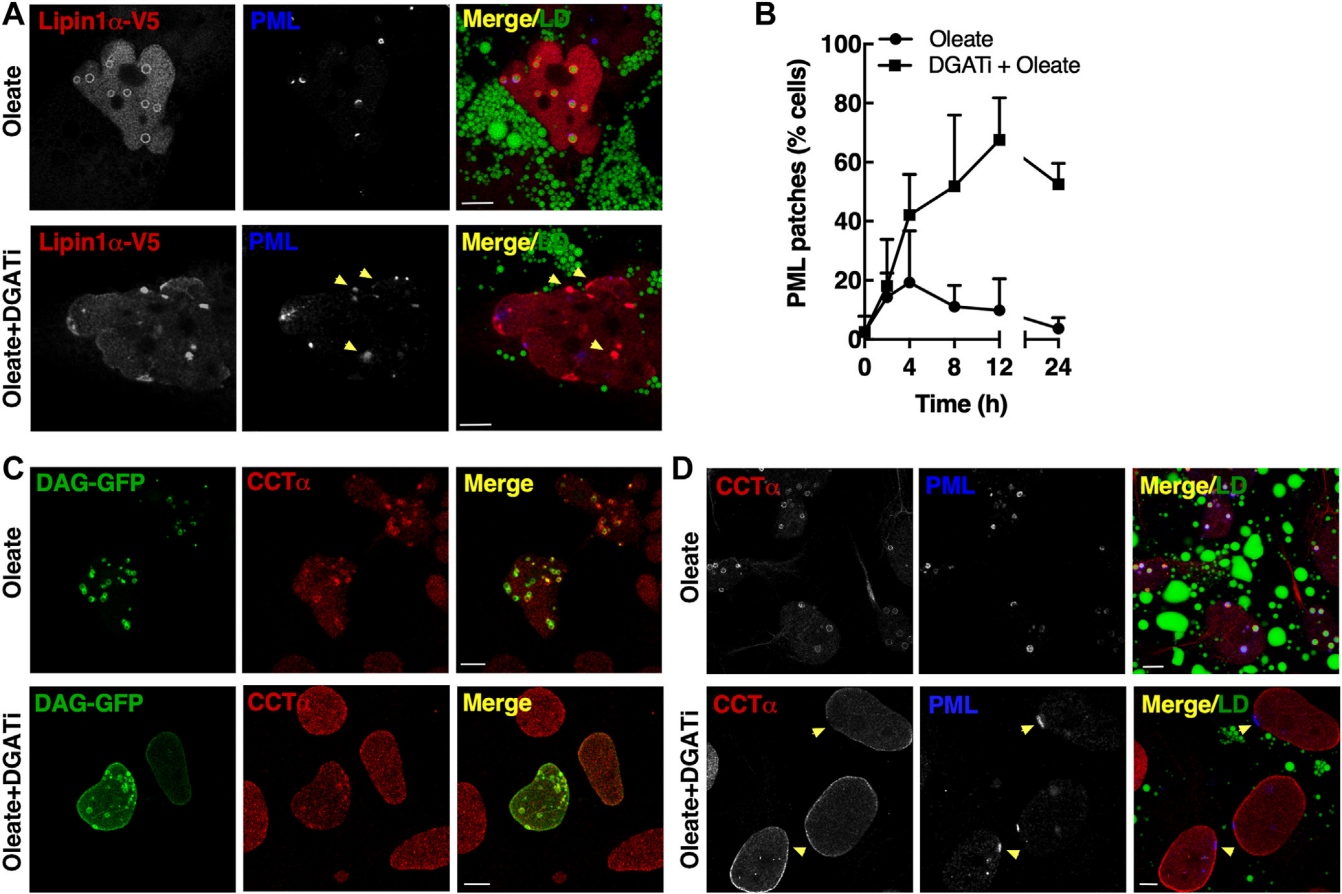
DGAT inhibition promotes retention of nLD- and LAPS-associated proteins on the INM of Huh7 cells. Huh7 cells were treated with oleate or oleate plus iDAGT1 and iDGAT2 as described in Fig. 9. A: Huh7 cells transiently expressing Lipin1α-V5 were immunostained with V5 and PML primary and AlexaFluor-555 and AlexaFluor-647 secondary antibodies, respectively. LDs were visualized with BODIPY493/503 (bar, 5 μm). B: the freqency of cells positive for PML patches was quantified after treatment with oleate or oleate plus DGATi for the indicated times using 15–18 fields of cells (30–60 cells/field) from two experiments. C: Huh7 cells transiently expressing a GFP-DAG biosensor were immunostained with a CCTα primary and AlexaFluor-555 secondary antibodies (bar, 5 μm). D: Huh7 cells were immunostained with CCTα and PML primary followed by AlexaFluor-555 and AlexaFluor-647 secondary antibodies. LDs were visualized with BODIPY 493/503 (bar, 5 μm). CCTα, CTP:phosphocholine cytidylyltransferase; INM, inner nuclear membrane; LAPS, lipid-associated promyelocytic leukemia structure; nLD, nuclear lipid droplet; PML, promyelocytic leukemia.

The effects of DGATi on Caco2 cells were compared to Huh7 cells, which have nLDs and LAPS derived from eLDs (13). Huh7 cells treated with oleate and DGATi had robust localization of Lipin1α−V5 to structures on the NE and in the nucleoplasm that partially localized with PML (see arrows in Fig. 10A). PML was localized to diffuse nuclear structures as well as patches on the NE of DGATi-treated Huh7 cells (see arrows in Fig. 10A, D). The frequency of PML patches on the NE in DGATi-treated cells increased to 70% by 12 h. Interestingly, oleate alone induced the formation of PML patches in 15%–20% Huh7 cells by 4 h, which declined it 5% by 24 h. In the presence of the DGATi, the GFP-DAG sensor localized on the NE and, in highly expressing cells, on nucleoplasmic structures that were in some cases positive for CCTα (Fig. 10C). In contrast to Caco2 cells, CCTα localized strongly to the INM in DGATi-treated Huh7 cells (Fig. 10C, D). The appearance of nLD- and LAPS-associated protein on the INM following inhibition of TAG synthesis in Caco2 and Huh7 cells points to this as the potential site for lipid synthesis required for nLD precursor expansion or in situ biogenesis.

## Discussion

nLDs were identified in oleate-treated Caco2 cells (18) and intestinal cells from *C. elegans* (6). However, the structure of nLDs, where and how they form and their role in intestinal fatty acid homeostasis is only partially elucidated. To address these questions, we utilized human Caco2 cells that can be differentiated into polarized monolayers with tight junctions and features of intestinal epithelial cells, including secretion of apoB48 chylomicrons (39). Immunofluorescence confocal microscopy showed that >80% of undifferentiated oleate-treated Caco2 cells contained nLDs and LAPS that harbored CCTα, Lipin1α, and DAG, features previously observed in U2OS cells and hepatocytes (4, 10, 13, 15). Caco2 cells differentiated on filter supports contained very large nLDs and LAPS (mean area 1–1.5 μm^2^) that formed irrespective of oleate treatment, did not associate with CCTα, and had small regions of associated PML due to their size. *C. elegans* intestinal cells also contain large nLDs (0.2–0.8 μm^2^) at different developmental stages that associate with heterochromatin, Type I NR, and the nuclear lamina (6). Intestinal nLDs in *C. elegans* were associated with the areas of NE damage and correlated with age-related nuclear deterioration (6), suggesting a pathophysiological role (40). Similarly, the accumulation of numerous large nLDs and LAPS in Caco2 cells is indicative of dramatic enhancement of nuclear TAG synthesis and metabolism as a consequence of oncogenic transformation.

nLDs and LAPS were present in human intestinal DLD1 and LS180 carcinoma cell lines as well as mouse enteroids, although much less frequently than Caco2 cells. Enterocytes in oleate-treated mouse intestinal enteroids had relatively few nLDs, which were identified based on their association with nuclear CCTα and lack of a surrounding nuclear lamina. A limitation of organoid culture is that the apical-luminal surface is facing inward. Thus, exogenous fatty acids are absorbed and re-esterified at the basolateral surface rather than the apical region, which is the physiological site for cLD biogenesis (41). The fact that enteroids have few nLDs suggests that an intrinsic capacity of these cells to divert TAG synthesis away from the INM is subverted by oncogenic transformation in some intestinal carcinoma cell lines.

A proposed function for nLDs and LAPS is to provide a platform for the translocation of CCTα and activation of PC synthesis, which counters ER stress caused by increased biogenesis of lipoproteins and cLDs (10, 13). Reduced PC synthesis in CCTα KO in Caco2 cells resulted in larger and fewer cLDs (30), consistent with the important role for PC in providing the cLD monolayer for TAG storage in LDs. CCTα KO caused a similar shift to fewer and larger total nLDs relative to controls in undifferentiated Caco2 cells, which argues that a common pool of PC is used to assemble cLDs and nLDs. Within the total nLD pool, CCTα deficiency favored the formation of LAPS possibly due to the preferred association of PML with large nLDs that were increased by CCTα deficiency or decreased surface crowding, a mechanism by which perilipin-3 displaces CCTα from the surface of nLDs and inhibits PC synthesis in Huh7 cells (13). CCTα was also identified on the surface of nLDs in mouse enteroid cells, but the relatively small number of nLDs suggest they play a minor role in CCTα regulation. The INM has a much greater surface area, and changes to INM lipid composition are responsible for CCTα activation in cells without nLDs (34, 42). With this in mind, it may be premature to assign a conserved role to nLDs in the regulation of PC synthesis.

The assembly of VLDL and chylomicrons in hepatocytes and intestinal epithelial cells involves the coalescence of an apoB-containing precursor particle with an eLD, which is dependent on MTP activity for its lipidation in the ER lumen (20, 43, 44). eLDs were identified as precursors for nLDs and LAPS in Huh7 cells based on reduced nLDs due to MTP inhibition and knockdown and the presence of apoE and apoCIII in nLDs (13). In contrast, there was limited evidence of an eLD/MTP-dependent pathway for nLD and LAPS assembly in undifferentiated Caco2 and other colon carcinoma cell lines cells, which had variable levels of nLDs and LAPS but very low MTP expression-relative Huh7 cells (Fig. 5). This agrees with prior studies showing that expression of apoB100, apoB48, and MTP in Caco2 cells occurs upon differentiation (38, 45, 46). MTP expression was increased during Caco2 differentiation as was the constitutive appearance of nLDs or LAPS. However, the maturation of nLDs in differentiating Caco2 cells was unaffected by MTP inhibition, and differentiated CCTα KO cells had abundant nLD and LAPS despite a profound block in chylomicron secretion (30) and a 70%–80% reduction in MTP expression (Fig. 4).

With limited evidence for a lipoprotein precursor origin for nLDs and LAPS, we explored an in situ mechanism for biogenesis at the INM. Time course and 3D imaging of nLDs and LAPS in undifferentiated Caco2 cells revealed a population of nascent particles that could serve as precursors. Similar primordial nLDs were observed at the INM of oleate-treated U2OS cells (15), and untreated Huh7 cells also contain 1-2 nascent nLDs and LAPS that increase 5-to 6-fold with oleate treatment (47). The number of nLDs and LAPS in Caco2 cells after exposure to oleate for 24 h was more than double that of the precursors indicating an additional mechanism(s) for biogenesis. This was not the case in differentiated Caco2 cells where the number of precursor and mature nLDs was similar. To identify where and how nLD assemble, we reasoned that early stages could be stalled and captured by inhibiting DGATs and TAG synthesis, thereby causing the accumulation of DAG and lipid biosynthetic enzymes at assembly sites. Inhibiting TAG synthesis with a combination of DGAT1 and 2 inhibitors did not affect precursor nLD or LAPS (Fig. 7C, D) but caused Lipin1α and the GFP-DAG sensor to be partially localized to the NE, a result also observed in Huh7 cells. Despite an increase in DAG at the INM of DGATi-treated Caco2 and Huh7 cells, only in Huh7 cells was CCTα localized to the INM. This discrepancy could be due to relative levels of DAG and fatty acids or another intrinsic feature of the INM in Caco2 cells that restricts CCTα translocation. Collectively, this points to the INM as the initial site for Lipin1α translocation and DAG synthesis and for activation of CCTα and PC synthesis in the case of Huh7 cells, followed by redistribution of the enzymes to nLDs and LAPS as they mature or bud from the INM. However, our inability to capture in situ biogenesis of nLDs or track the expansion of precursors by live cell imaging in Caco2 cells leaves the precise mechanism(s) open to question.

PML-NBs redistribute to LAPS in Caco2 cells where they constitute 20%–40% of the total nLDs and become deSUMOylated. Caco2 cells contain a small number of pre-existing LAPS, and after brief oleate treatment (2 h), PML was associated with small nLDs in proximity to the INM. Blocking biogenesis with DGATi for 4–8 h caused PML to redistribute to a variety of structures including patches on the INM, filaments, and diffuse bodies in Caco2 and Huh7 cells. PML patches were previously observed in oleate-treated Huh7 cells and proposed to link nLDs and the NE to chromatin (4). These unusual PML-containing nuclear subdomains formed by inhibition of TAG synthesis also partially overlapped with DAG, Lipin1α, and CCTα. We propose that these PML structures are precursors to LAPS assembly and have been captured by the inhibition of nLD formation and resultant abnormal lipid composition of nuclear membranes. The presence of PML, CCTα, Lipin1α, and DAG on the INM of DGATi-treated Huh7 cells, which have an eLD–MTP pathway that accounts for 50%–60% of nLD biogenesis in oleate-treated cells (13), suggests an in situ pathway could also be active.

Intestinal epithelial cells experience periodic and acute influx of dietary fatty acids that are re-esterified and packaged into cLDs and secreted in chylomicrons. Modeling this system in Caco2 cells, we show that excess mono-unsaturated fatty acids or differentiation drive the formation of nLDs and LAPS by precursor expansion and in situ mechanisms at the INM through recruitment of Lipin1α and CCTα for the synthesis of TAG and PC components of lipid droplets. nLDs and LAPS are highly abundant in Caco2 cells compared to other intestinal cells and mouse enteroids, a manifestation of abnormal TAG synthesis and storage due to oncogenic transformation. A similar diversion of TAG to nLDs occurred in a mouse liver knockout of the lamin-associated protein 1, which caused steatosis and accumulation of large nLDs due to inhibition of VLDL secretion (3, 48). Hepatic nonalcoholic steatosis is also reported to increase the abundance of nLDs (49). Similarly, our studies describe how nLD assemble in Caco2 cells; however, whether this applies to normal cells, and the pathological mechanisms that promote accumulation of these novel lipid stress-induced nuclear subdomains, is still unknown.

## Data availabilty

All data is contained in this manuscript.

## Supplemental data

This article contains supplemental data.

## Conflict of interest

The authors declare that they have no conflicts of interest with the contents of this article.
