# Supplementary material for: Nuclear lipid droplets in Caco2 cells originate from nascent precursors and in situ at the nuclear envelope
Source: J Lipid Res. 2024 Apr 1;65(5):100540. doi: 10.1016/j.jlr.2024.100540 (PMC11077042; doi:10.1016/j.jlr.2024.100540)
Supplement: Supplemental Data [file mmc1.pdf]

Supplemental Figure 1

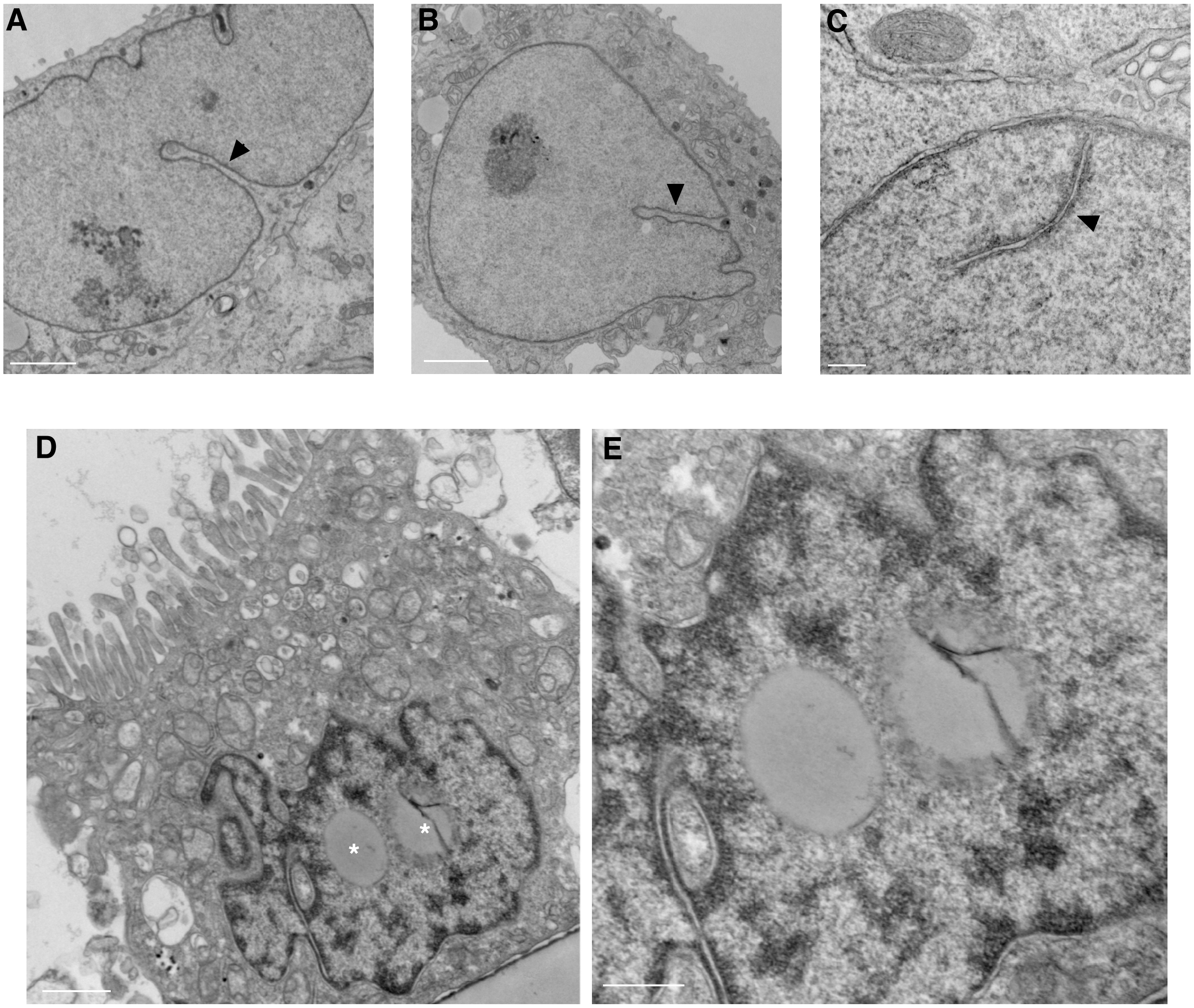

Supplemental Figure 1. **Transition electron micrographs of undifferentiated and differentiated Caco2 cells.** A-C, TEM images of undifferentiated Caco2 cells treated with oleate (500 μM) for 8 h showing Type II (panels A and B, bar 2 μm) and Type I (panel C, bar 200 nm) nucleoplasmic reticulum (indicated by arrows). D, differentiated Caco2 cells have well-defined apical microvilli and multiple large nLDs indicted by asterisks (bar, 1 μm). E, magnified field from panel D showing nLDs (bar, 400 nm).

Supplemental Figure 2

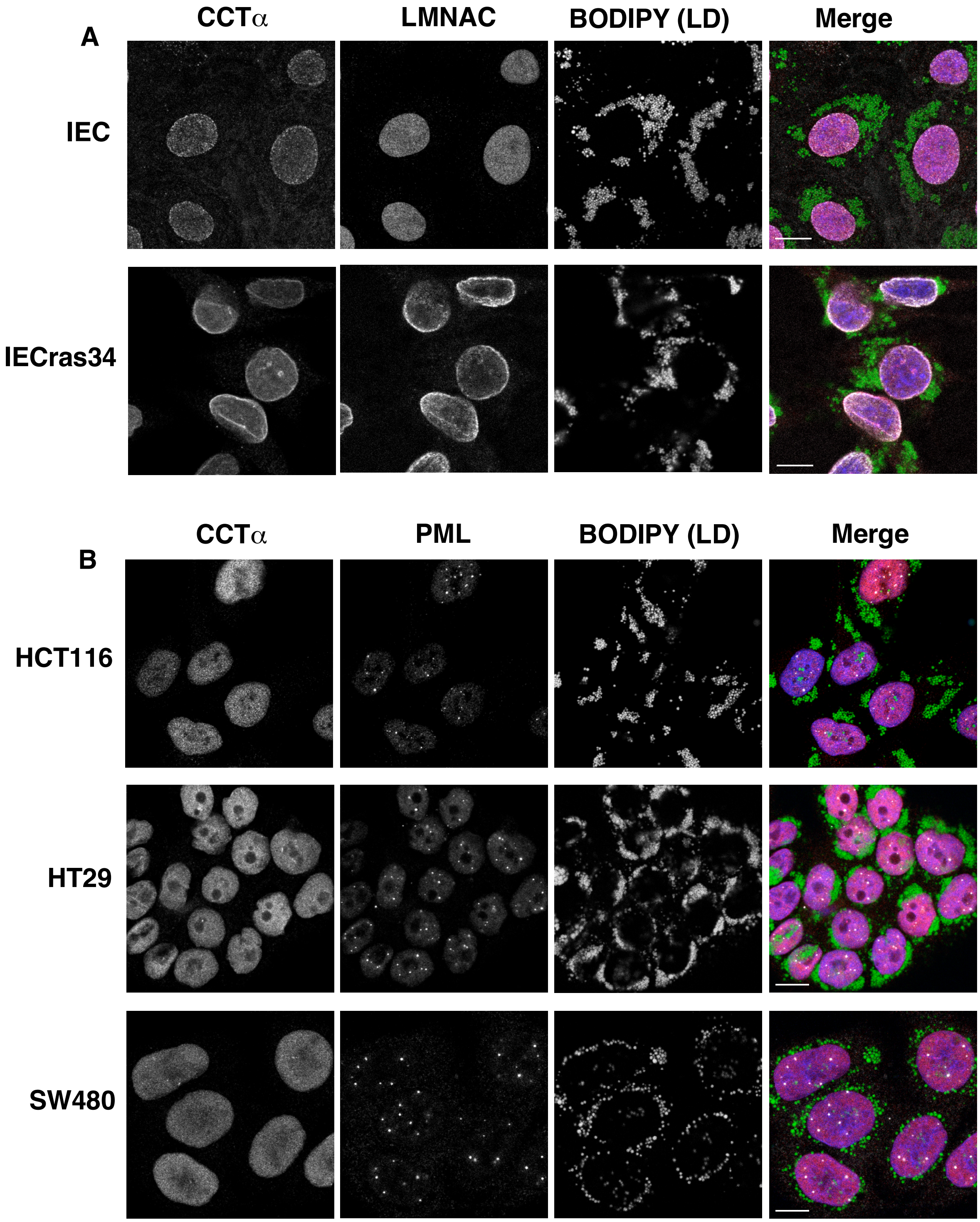

Supplemental Figure 2. **IEC18, IECras34, HCT116, HT29 and SW480 cells are devoid of nLDs and LAPS.** A, IEC and IECras34 cells incubated with oleate (500 μM) for 24 h were immunostained with CCTα and LMNA/C primary and AlexaFluor-494 and -647 secondary antibodies, respectively. LDs were visualized with BODIPY493/503 (bar, 10 μm). B, HCT116, HT29 and SW480 cells incubated with 500 μM oleate for 24 h were immunostained with CCTα and PML primary and AlexaFluor-494 and -647 secondary antibodies, respectively. LDs were visualized with BODIPY493/503 (bar, 10 μm).

Supplemental Figure 3

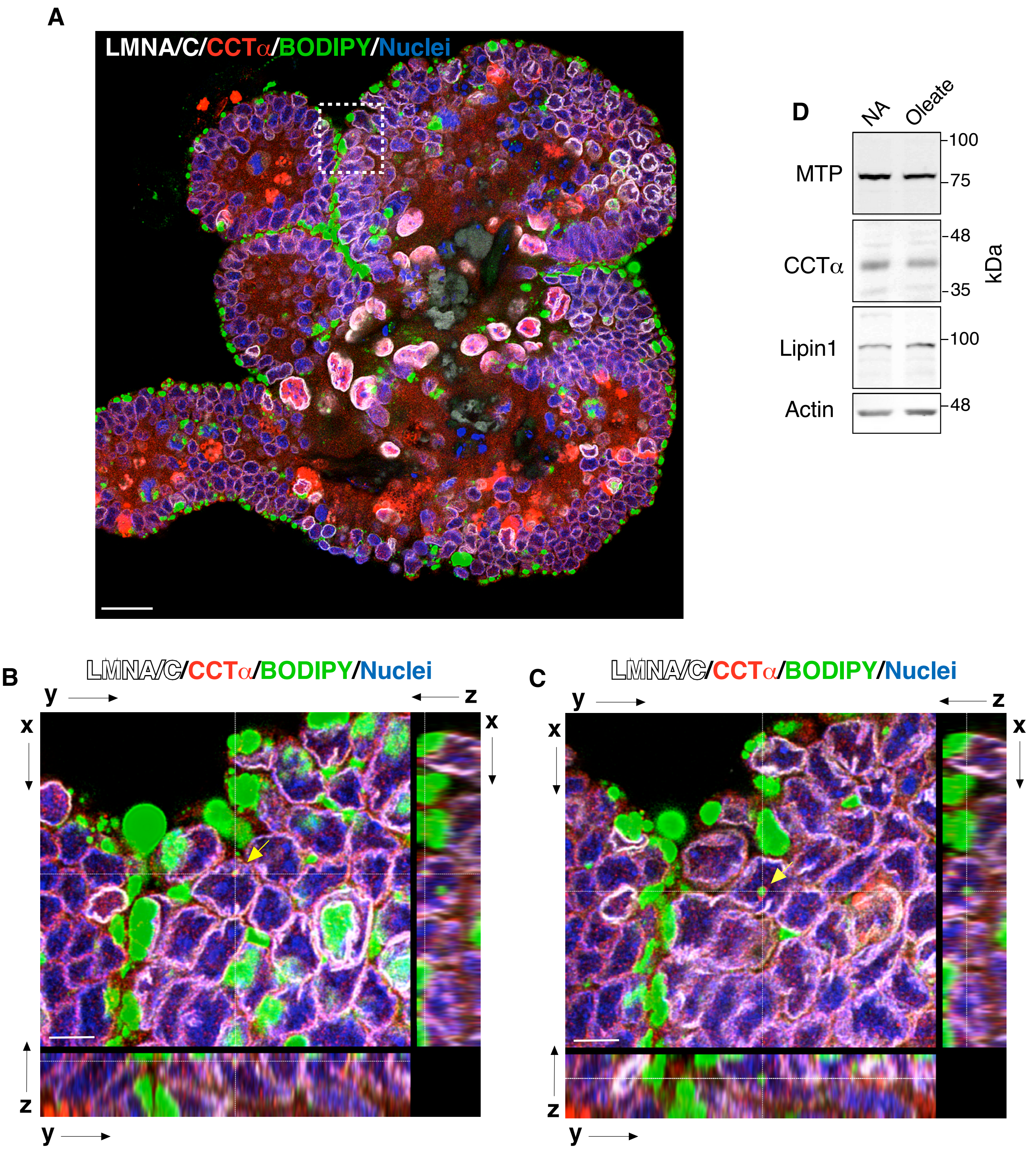

Supplemental Figure 3. **Confocal imaging of a mouse enteroids.** A, enteroids were immunostained for CCTα and LMNA/C, LDs were stained with BODIPY 493/503 and the nuclei with Hoechst (bar, 20 μm). B and C, additional xyz-projections of the region outlined in panel A showing CCTα-positive nLDs (indicated by arrows) in the nucleus of enterocytes (bar, 5 μm). D, whole enteroids were lysed and immunoblotted for the indicated proteins (actin load control).

Supplemental Figure 4

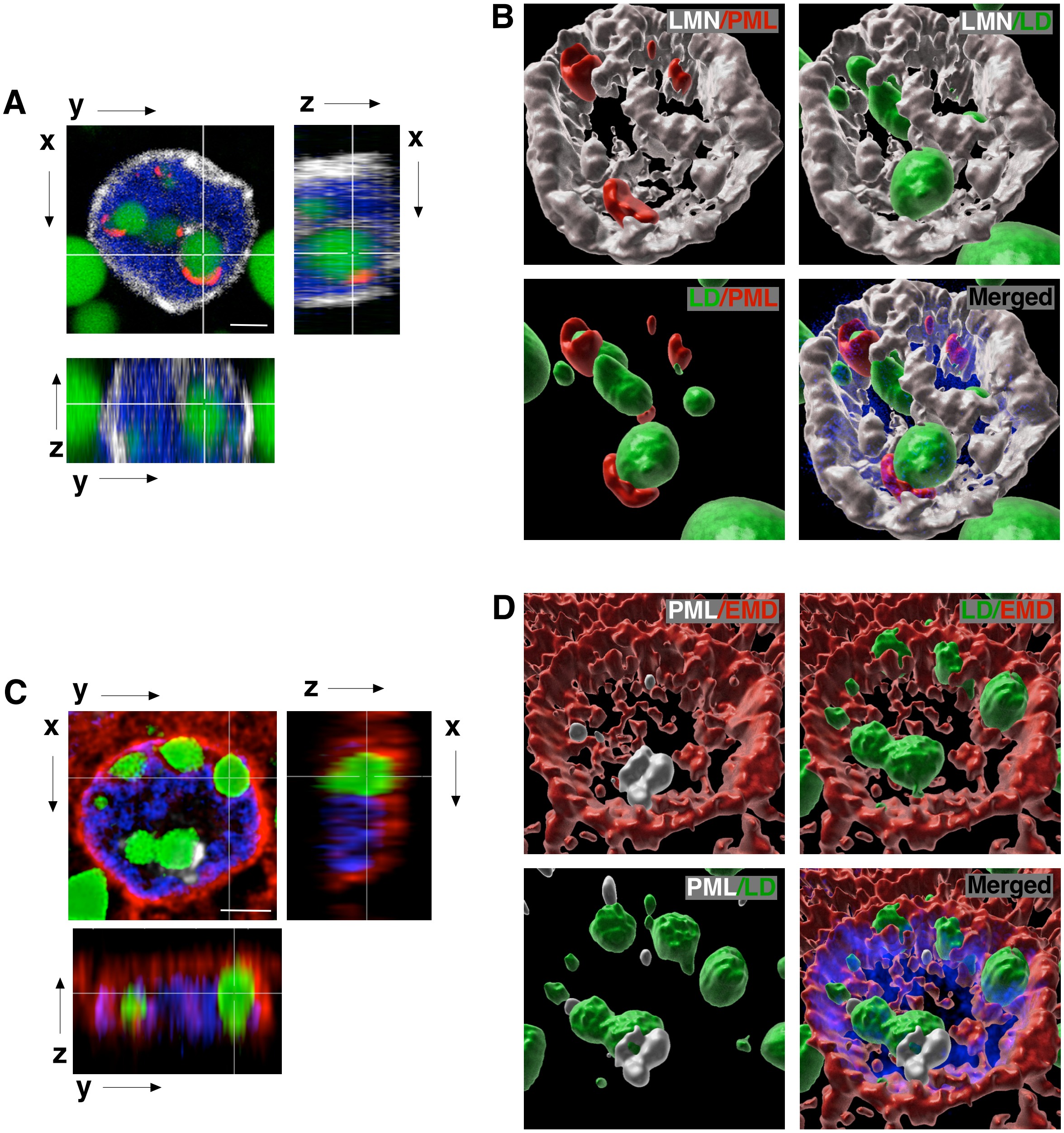

Supplemental Figure 4. **Confocal imaging and 3D reconstructions of nLDs and LAPS in differentiated Caco2 cells.** Cells were treated and processed for immunofluorescence microscopy of LNMA/C, emerin (EMN) and PML as described in the legend to Figure 7 (bar, 2 μm). 3D images in panels B and D are rotated slightly to the left relative to the x-y images in panels A and C.
